# Recent advances in tendon tissue engineering strategy

**DOI:** 10.3389/fbioe.2023.1115312

**Published:** 2023-02-20

**Authors:** Chao Ning, Pinxue Li, Cangjian Gao, Liwei Fu, Zhiyao Liao, Guangzhao Tian, Han Yin, Muzhe Li, Xiang Sui, Zhiguo Yuan, Shuyun Liu, Quanyi Guo

**Affiliations:** ^1^ Chinese PLA Medical School, Beijing, China; ^2^ Beijing Key Lab of Regenerative Medicine in Orthopedics, Key Laboratory of Musculoskeletal Trauma and War Injuries PLA, Institute of Orthopedics, Chinese PLA General Hospital, Beijing, China; ^3^ Department of Bone and Joint Surgery, Renji Hospital, School of Medicine, Shanghai Jiaotong University, Shanghai, China

**Keywords:** tendon tissue engineering, biomaterial scaffolds, cells, biological adjuncts, mechanical stimuli, macrophage polarization

## Abstract

Tendon injuries often result in significant pain and disability and impose severe clinical and financial burdens on our society. Despite considerable achievements in the field of regenerative medicine in the past several decades, effective treatments remain a challenge due to the limited natural healing capacity of tendons caused by poor cell density and vascularization. The development of tissue engineering has provided more promising results in regenerating tendon-like tissues with compositional, structural and functional characteristics comparable to those of native tendon tissues. Tissue engineering is the discipline of regenerative medicine that aims to restore the physiological functions of tissues by using a combination of cells and materials, as well as suitable biochemical and physicochemical factors. In this review, following a discussion of tendon structure, injury and healing, we aim to elucidate the current strategies (biomaterials, scaffold fabrication techniques, cells, biological adjuncts, mechanical loading and bioreactors, and the role of macrophage polarization in tendon regeneration), challenges and future directions in the field of tendon tissue engineering.

## 1 Introduction

As the most common disorders in the musculoskeletal system, tendon injuries affect millions of people and present a serious problem to society (Steinmann et al., 2020). According to the literature recordings, the rotator cuff, Achilles tendon and patella tendon are the most common injury sites (Butler et al., 2004). The etiology of tendon injuries is multifactorial and includes trauma, chronic overuse, aging, inflammation and genetic factors (Liu Q. et al., 2021). Owing to their hypocellular and hypovascular nature, tendons exhibit a limited regenerative capacity, and the natural repair process results in fibrotic scar tissue with inferior mechanical properties (Nourissat et al., 2015; Hou et al., 2021). After injury, the tendon needs extended time to rehabilitate its structure and mechanical functions.

**FIGURE 1:**
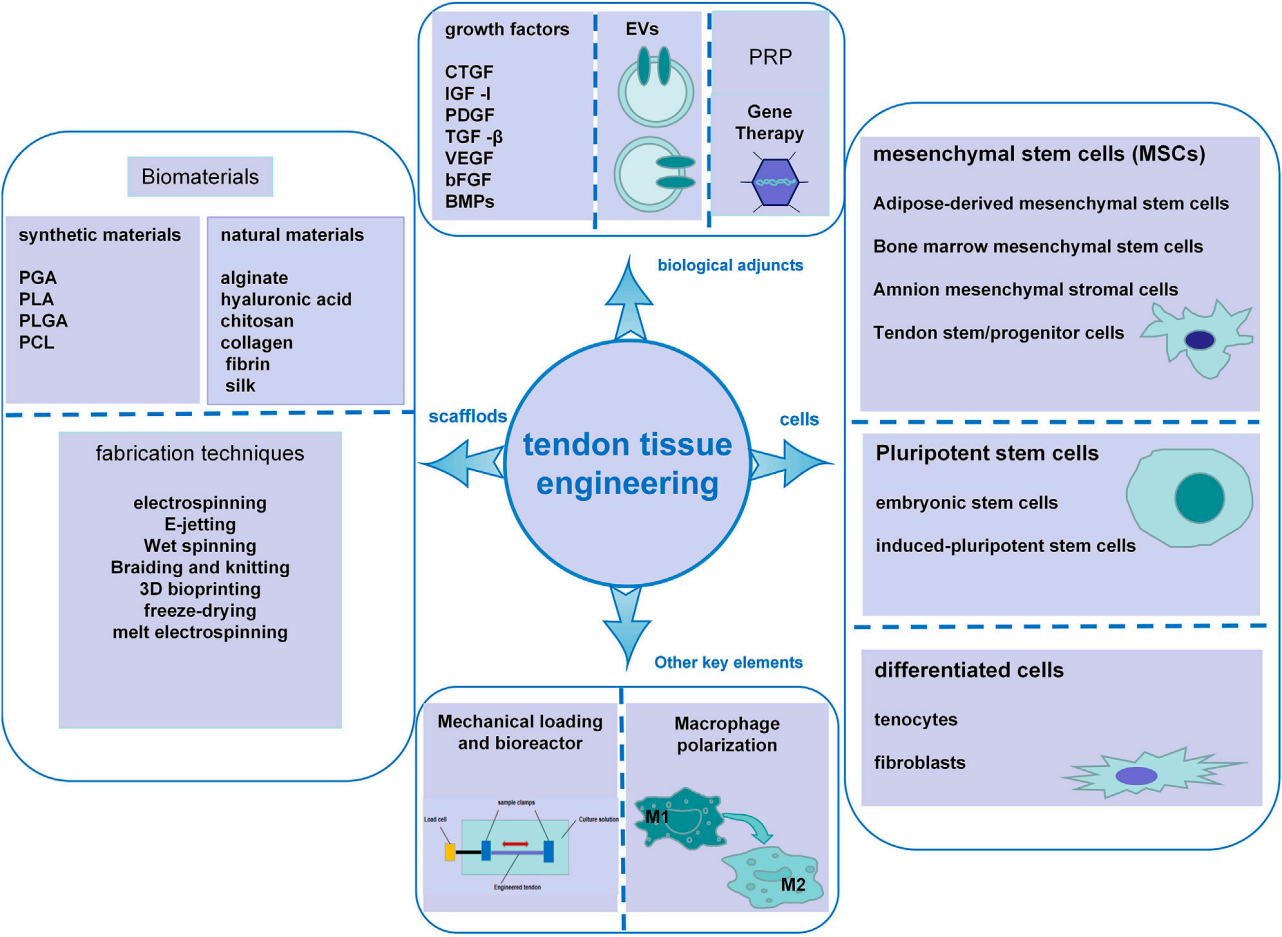
Schematic illustration of the key elements in tendon tissue engineering, including scaffold biomaterials and fabrication techniques, cells, biological adjuncts, mechanical loading and bioreactors, and macrophage polarization.

Currently, conservative treatments and surgical treatments (direct suture, transplantation of autografts, allografts and tendon prostheses made of inert synthetic materials) are the main therapies for tendon defects. Other emerging treatments involving the infiltration of cells or growth factors have also been reported (Leong et al., 2020). Different methods have achieved some positive results; however, each method has certain advantages and disadvantages (Salamon et al., 1970; Beris et al., 2003). Thus, designing an effective and lasting resolution is imperative. With increasing knowledge about tendon biology, pathology, and healing and advances in biomaterials involving their fabrication techniques, biological adjuncts and other associated elements, tissue engineering has emerged as a promising approach for musculoskeletal tissue repair and regeneration by using a combination of biomaterials, cells and/or growth factors to recover, maintain or improve structure and function by mimicking the native tissues (Font Tellado et al., 2015; Ruiz-Alonso et al., 2021). In this review, we discuss the biological structure of tendons, their injury and healing processes, and clinical treatments, and we present the progress in tissue engineering methods involving materials, scaffold fabrication techniques, cells, biological adjuncts and other relevant elements. We also discuss current challenges and future directions to provide references for the development of new and more efficient therapies to manage tendon injuries (Figure 1).

## 2 Structure and composition of tendon

**FIGURE 2:**
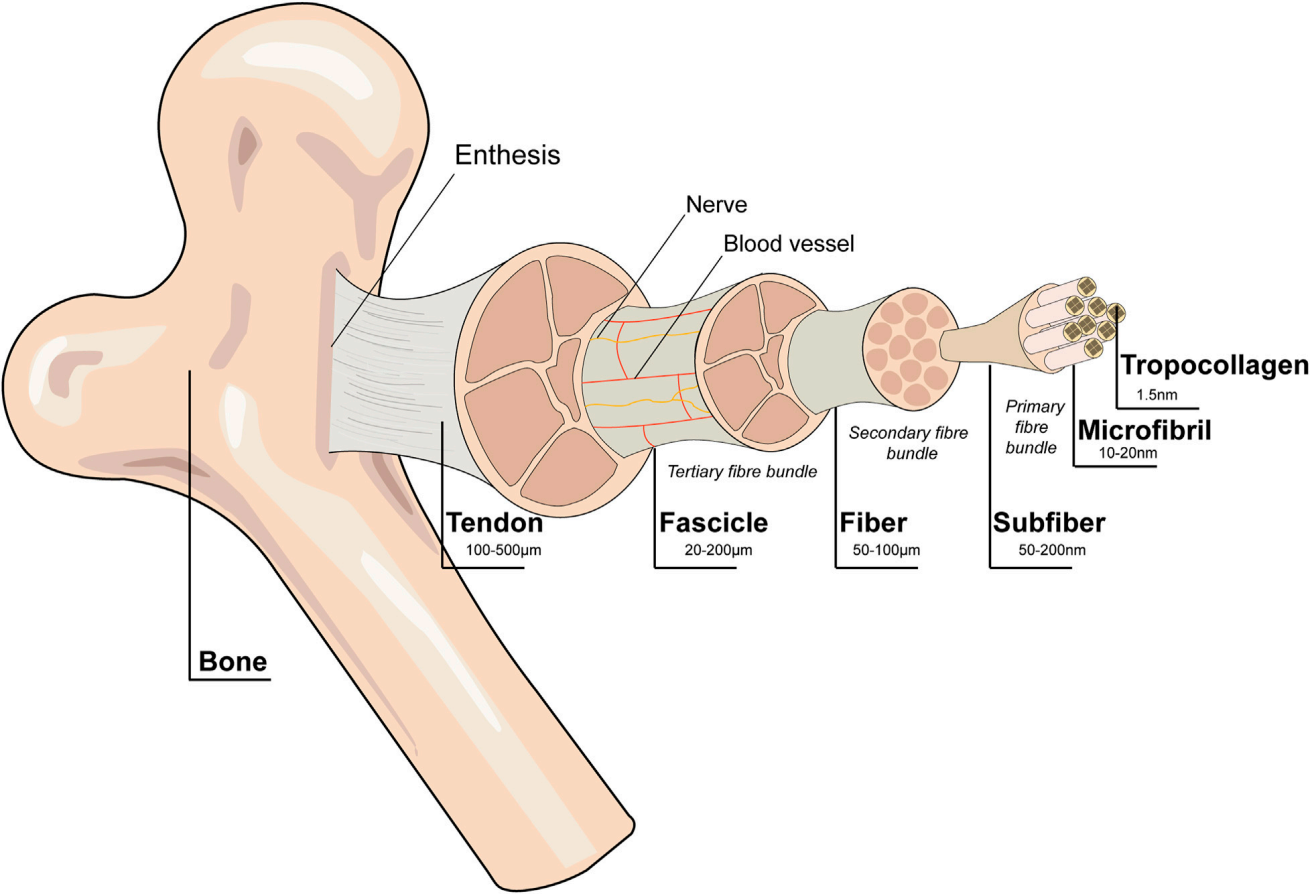
Schematic diagram of the tendon hierarchical structure. Collagen alignment is based on organization into fascicles, fibers, and fibrils. Tenocytes are the main cellular component of tendons. Blood vessels and nerves are present within the structure. Reproduced with permission (Ruiz-Alonso et al., 2021).

Tendons consist of a hierarchically organized complex fibrous connective tissue (Figure 2) whose functions involve connecting muscles to bones, translating muscle contractile forces into body movement, and reducing the stress concentration between muscle and bone by providing appropriate stiffness. Due to the critical role of this tissue in body mechanics, the injury and degeneration of tendons can result in substantial pain, disability, and healthcare costs (Voleti et al., 2012).

Tendons are composed of extracellular matrix (ECM) and cells. The ECM is composed predominantly (roughly 70% by dry weight) of type I collagen and small amounts of elastin, proteoglycans and glycoproteins (Voleti et al., 2012). The ECM not only serves as the microenvironment for cells to reside but also initiates important biochemical and biomechanical cues to maintain tissue morphogenesis, differentiation and homeostasis (Frantz et al., 2010). Tenocytes and tendon progenitor/stem cells account for approximately 90%–95% of tendon cells. The remaining 5%–10% of tendon cells are chondrocytes, synovial cells of the tendon sheath, capillary endothelial cells and arteriolar smooth muscle cells (Schneider et al., 2018). Tenocytes are specialized fibroblast cells with an elongated form and are stellate in cross section (Ruiz-Alonso et al., 2021). Tenocytes distributed in the endotenon area are termed interfascicular tenocytes, while the cells distributed in the fascicle area are termed interfascicular tenocytes (Zhang et al., 2022). Tenocytes regulate tendon ECM remodeling by producing growth factors, type I collagen and associated ECM molecules (Andarawis-Puri et al., 2015). Tendon stem cells (TSCs) have been isolated in cell cultures from human, rabbit, rat and mouse tendons and have regenerative properties different from those of bonemarrowderived mesenchymal stem cells (MSCs) (Bi et al., 2007; Zhang and Wang, 2010a). Tendon stem cells have been applied in tissue engineering to foster the repair and regeneration of tendon tissue (Zhang et al., 2020). When tendons transfer mechanical stimuli to resident cells, the ECM provides tensile strength (Wang et al., 2012). The interaction between cellular behavior and ECM is vital to maintain the homeostasis and normal function of tendons.

As the basic unit of tendons, fibrils consist of collagen molecules, range from a few nanometers to approximately 500 nm in diameter, and exhibit a bimodal distribution to counteract fibril slip and tensile strength (Birch et al., 2013; Zhang et al., 2022). Collagen fibrils gather to form collagen fibers and then to form fascicles. Fascicles, the basic unit of tendons, are irregular in shape and vary in diameter, ranging from 150 to 500 µm. Endotendinous connective tissue (endotenon) is a kind of connective tissue compartment that encompasses fascicles and contains blood vessels and nerves. Tendons consist of numerous fascicles surrounded by a connective tissue sheath called the epitenon (Zhang et al., 2022). This hierarchical organization imparts high viscoelasticity, non-linear elasticity and anisotropy (Ruiz-Alonso et al., 2021; Wang et al., 2021).

## 3 Tendon injury and healing

Tendon injury modalities involve acute or chronic changes or their combination. Acute injuries are often associated with excessive or absent mechanical loading in sports activities, while chronic injuries are mainly related to degeneration and inflammation with histological changes, including enhanced cellularity, vascularity, and collagen misalignment (Hyman and Rodeo, 2000). When the tendon is injured, its structure and function can be compromised. Due to their hypocellularity, poor vascularity and low metabolic activity, the injured tendon can rarely return to the natural structure and morphology but instead exhibit scar tissue formation with some degree of inferior mechanical function (Docheva et al., 2015). Thus, the mechanical properties of the healed tendon are not comparable with those of the original healthy tissue, which results in a high percentage of rupture (Riley, 2004).

There are two different healing types, intrinsic and extrinsic patterns. Mild injuries with the epitenon intact lead to intrinsic healing, which is characterized by superior mechanical properties and fewer complications (Voleti et al., 2012). Epitenon injuries result in extrinsic healing with severe complications (Yang et al., 2013). There are three overlapping stages in the extrinsic healing process: the inflammatory stage, characterized by hematoma formation and inflammatory cell infiltration; the proliferative or repair stage, characterized by profuse synthetic activity directed by macrophages and tenocytes releasing growth factors and depositing ECM; and the remodeling stage, characterized by decreased cell density and better mechanical properties of the ECM with a more aligned structure. All three overlapping stages can last more than 1 year (Voleti et al., 2012). There are no clear boundaries between the three stages, and we do not currently understand the full complexity of the natural healing process.

## 4 Clinical treatment methods

### 4.1 Non-surgical treatments

In the acute phase, initial rest combined with braces and immobilization are the most important treatment methods (Irwin, 2010). Non-invasive training and exercises are necessary during treatment to avoid the complications caused by prolonged immobilization. As a commonly used physical therapy, ultrasound can reduce swelling caused by the inflammatory response and promote collagen synthesis and cell division to enhance tendon healing (Best et al., 2015). Low-level laser therapy (LLLT) is another effective treatment method that reduces the inflammatory response, stimulates tenocyte proliferation, decreases the capillary flow of neovascularization, increases collagen production, and preserves the resistance and elasticity of tendons. Extracorporeal shockwave therapy can also facilitate tendon healing by promoting proinflammatory and catabolic processes. Although it is clear that NSAIDs can relieve pain in the acute phase, whether their overall effects on tendon repair and healing are favorable remains controversial. It has been demonstrated that corticosteroids can reduce pain and swelling. However, the adverse effects, including tendon atrophy, tendon rupture and decreased tendon strength, cannot be ignored (Li and Hua, 2016).

### 4.2 Reparative treatments

Currently, we have some alternative therapies for tendon repair, including biological grafts (autografts, allografts and xenografts), permanent artificial prostheses, and tissue engineering. Biological grafts have several shortcomings, including donor site morbidity, limited availability, disease transmission and immunological rejection. Permanent artificial prostheses lack material durability and often lead to mechanical failures later on. The emergence of tissue engineering technology has provided us with a new choice to cope with tendon injuries effectively and completely.

### 4.3 Regenerative treatments

Tissue engineering involves the use of a combination of cells, scaffolds and biologically active molecules (Berthiaume et al., 2011). Cells serve as seeds of the repaired or regenerative organ. Scaffolds provide a 3-D template and mechanical stability to facilitate tissue regeneration and provide a microenvironment for cells and biologically active molecules that act as drivers for cell attachment, proliferation, migration and differentiation (Lim et al., 2019). In the following sections, a comprehensive summary of tendon tissue engineering advances will be discussed, including different aspects such as strategies involving scaffolds, stem cells, biological adjuncts, mechanical loading and bioreactors, the role of macrophage polarization, current challenges and future directions.

## 5 Strategies involving scaffolds for tendon tissue engineering

Scaffolds serve as a temporary three-dimensional construct with an appropriate microenvironment mimicking the structure and function of the native tendon and degrade gradually as the regenerated tissue forms. The key factors in designing scaffolds are porous structures with varied pore sizes should be biocompatible with no or little immune rejection by the body; the degradation rate should be comparable to the regeneration rate of tendon tissue; and the degradation products should be harmless (Liu Q. W. et al., 2021); excellent mechanical properties and porous structure: the scaffolds should have excellent mechanical properties to withstand repeated stresses and forces, the scaffolds should have porous structures to facilitate nutrient and waste transport, cell attachment, proliferation and in-growth (Nourissat et al., 2015); economical and versatile fabrication methods: the scaffold fabrication techniques should be convenient and versatile to enable the widest possible application (Howard et al., 2008). The biomaterials and fabrication techniques of scaffolds are the two main elements that require focus.

### 5.1 Biomaterials used for tissue engineering scaffolds

The ideal biomaterials for scaffold construction should have the following properties: superior mechanical strength throughout the tissue regeneration process; excellent biocompatibility with the surrounding tissues; biodegradability with an adjustable degradation rate; good biofunctionality to support cell proliferation, differentiation, ECM secretion and tissue formation; flexible processability to form desired structures; and hydrophilicity and wettability to support cell adhesion (Liu et al., 2008; Lim et al., 2019). To date, the commonly used biomaterials in tissue engineering include natural materials and synthetic biomaterials. The advantages and disadvantages of the commonly used polymers for tendon tissue engineering are summarized in Table 1.

**TABLE 1:** Overview of some commonly used natural and synthetic polymers for tendon tissue engineering.

Polymer type	Polymer name	Advantages	Disadvantages	References
Natural polymers: Polysaccharide	alginate	Low-cost utilization, biocompatibility, low toxicity, structural similarity to ECM and gelling capacity	Poor mechanical properties, poor cell adherence	Hou et al. (2021), Abourehab et al. (2022), Hurtado et al. (2022)
	hyaluronic acid	ECM components, biocompatibility, mucoadhesivity, hygroscopicity, viscoelasticity, anti-inflammatory and anti-adhesive responses	Poor mechanical properties	Kaux et al. (2015), Han et al. (2019), Oliva et al. (2021), Ruiz-Alonso et al. (2021)
	chitosan	Ease of modification, antibacterial and antioxidant activity, non-toxicity, biocompatibility, and biodegradability	Low mechanical properties, limited cell adhesion properties	Muxika et al. (2017), Kulkarni et al. (2021)
Natural polymers: Protein-based materials	collagen	ECM components, low inflammatory and immunogenic responses, natural abundance, biodegradability, cell recognition, controllable mechanical properties	Rapid degradation kinetics, poor structural stability, potential immunogenicity	Citeroni et al. (2020), Citeroni et al. (2020)
	fibrin	Biocompatibility and biodegradability, elastic and viscous properties, high seeding efficiency, adhesion capabilities and uniform cell distribution	Easy shrinkage and poor mechanical stability, rapid degradation	Ahmed et al. (2008), Park and Woo (2018), Roberts et al. (2020); Rojas-Murillo et al. (2022)
	silk fibroin	Excellent biocompatibility, biodegradability, bioresorbability, low immunogenicity, high mechanical strength and tunable mechanical properties, low immunogenicity	Poor cell adherence and growth, low biodegradability	Cao and Wang (2009), Kundu et al. (2013), Yao et al. (2016)
Synthetic polymers	PGA, PLA and PLGA	Biocompatibility, biodegradability, good mechanical properties and excellent processability	Low bioactivity, poor cellular adhesion and growth, acidic degradation products eliciting inflammatory response	Araque-Monrós et al. (2020), Mao et al. (2021), Rinoldi et al. (2021)
	PCL	Good processability, biodegradability, and biocompatibility, low melting temperature, excellent mechanical resistance, and relatively low cost	Hydrophobicity, inadequate wettability, lack of bioactive functional groups	Backes et al. (2022), Lim et al. (2021)

#### 5.1.1 Natural materials

Because of their ease of derivatization, excellent biocompatibility, biodegradability, favorable cell adhesion, and other biochemical cues, natural materials are widely used in tissue engineering (Berthiaume et al., 2011). They can be divided into two different categories, polysaccharide materials (alginate, hyaluronic acid, chitosan) and protein biomaterials (collagen, fibrin, silk) (Hou et al., 2021). Most of these materials have some drawbacks, including poor mechanical properties, uncontrollable degradation time and large batch-to-batch variation, which limit their clinical application (Sun et al., 2021).

##### 5.1.1.1 Polysaccharide materials

Polysaccharide materials readily form loose viscoelastic gels in aqueous vehicles *via* non-covalent interactions (Tiwari and Bahadur, 2018). They have similar biological constituents to the ECM. Alginate (ALG) and hyaluronic acid (HA) are highly biocompatible polyanions with high charge density, hydrophilicity, and functional groups analogous to those on GAGs (Hou et al., 2021).

Alginate is one of the naturally abundant and anionic polysaccharides composed of the disaccharide repeating unit [GlcAβ(1–4)Glcβ(1–3)], implying its low cost utilization. This anionic polymer is characteristic of biocompatibility, low toxicity, structural similarity to ECM and gelling capacity using different methods including covalent crosslinking, ionic crosslinking and thermal gelation (Abourehab et al., 2022). However, alginate hydrogels possess very weak mechanical properties, especially when they are hydrated in water, and have very low electrical and thermal conductivity, non-antibacterial activity, and very poor cell binding activity (Hurtado et al., 2022). To deal with the poor mechanical properties, alginate is often use in combination with other biological materials (chitosan, polylactic-co-glycolic acid, *etc.*) to form composite scaffolds (Ruiz-Alonso et al., 2021). Moreover, in order to cope with the poor cell adherence due to the lack of a cell binding motif, immobilization of the alginate peptide is an alternative to promote cell adherence and prevent cell apoptosis. Yao et al. (2022) utilized polycaprolactone/sodium alginate hydrogel scaffolds loaded with melatonin to facilitate tendon repair by activating the Nrf-2/HO-1 signaling pathway. In addition to the repair effect, alginate can also be coated on braided scaffolds to prevent peritendinous adhesion (Jayasree et al., 2019). Namba et al. (2007) also showed that alginate can have an antiadhesive effect and promote tendon repair.

HA is composed of repeating units of N-ace-tyl-D-glucosamine and D-glucuronic acid and forms the molecular backbone of proteoglycan complexes (Chen J. et al., 2021). As one of the fundamental components of tendon tissue, HA contribute viscoelastic properties to tendons. HA has been used to promote tendon healing by the properties of biocompatibility, mucoadhesivity, hygroscopicity, and viscoelasticity (Oliva et al., 2021). HA is also characteristic of anti-inflammatory properties by promoting macrophage of M1 phenotype differentiation into the M2 phenotype and lubrication properties by inhibiting fibroblast proliferation and reducing production of type III collagen concentration (Kaux et al., 2015). HA regulates cellular viability, adhesion, migration and proliferation to facilitate tendon repair and involved in the repair process by modulating inflammation, cellular migration, and angiogenesis (Abatangelo et al., 2020). Many literatures have reported the favorable effects of HA for tendon repair. Sun et al. (2004) and Nishida et al. (2004) both demonstrated that HA may improve the gliding function of flexor tendons *in vitro*. In addition, the injection of high molecular weight hyaluronan was effective for pain relief and repair promotion in a tendinopathy model (Yoshida et al., 2015). Frizziero et al. (2015) also demonstrated that HA can maintain the structural properties of the patellar tendon and enthesis in detrained rats. Although with the favorable effects, the utilization of HA is often compromised by the disadvantages of the low mechanical properties. The solution is to use it in combination with other materials for creating scaffolds withe more complex and better mechanical properties (Han et al., 2019; Ruiz-Alonso et al., 2021).

Chitosan (CS) is a series of chitin-derived cationic polymers with bioactive properties and can be used in tendon tissue engineering alone or combined with other polymers. The structure of CS is similar to that of native extracellular proteoglycans (Hameed et al., 2022). The positive charge in chitosan allows for many electrostatic interactions with negatively charged molecules. CS has advantages in biomedical applications due to its ease of modification, antibacterial and antioxidant activity, non-toxicity, biocompatibility, and biodegradability (Muxika et al., 2017). CS solubility is pH responsive and depends on the distribution of free amino and N-acetyl groups. Chitosan is hypoallergenic and only transiently stimulates the immune system, then becomes biotolerated and metabolized when impregnated with tissue fluids in the receptor body. Chitosan can be manufactured into a porous structure with controllable porosity to support cell proliferation, migration, exchange of nutrients and angiogenesis of the regenerated soft tissues. The application of CS in tendon tissue engineering is often compromised by the low mechanical properties and limited cell adhesion properties (Kulkarni et al., 2021). In order to cope with the drawbacks, CS is often dealed with chemical modification or combined with natural and synthetic materials to form CS-based materials including 3D lyophilized scaffolds, hydrogels and films (Muxika et al., 2017) to promote mechanical properties and cell affinity (Huang et al., 2009). Chitosan/gelatin-tannic acid (CS/GE-TA)-decorated sutures exhibit enhanced mechanical properties, superior anti-inflammatory and antibacterial performance, and increased collagen deposition and blood vessel formation (Zhang H. et al., 2021). And that, a zinc oxide (ZnO) nanoparticle-loaded CS scaffold enhanced gliding function and histopathological expression and reduced adhesion formation in the rabbit deep digital flexor tendon repair process (Yousefi et al., 2018).

##### 5.1.1.2 Protein biomaterials

As the main component of the ECM, collagen can support cell growth and maintain the structural and biological integrity and mechanical resilience of connective tissues. As the native components of tendon, collagen molecules gather to form fibrils and then fibers, and further form the basic structure of tendon (Sorushanova et al., 2019). Collagen is widely used in tissue engineering due to its properties of natural abundance, biocompatibility, biodegradability, cell recognition, controllable mechanical properties, various physical conformations (Citeroni et al., 2020). In order to promote the repair effects, collagen is often combined with other elements to form a composite scaffold for tendon tissue engineering. Collagen scaffolds can combine with different cells, growth factors or platelet-rich plasma to promote tendon repair or regeneration (Kew et al., 2011). And that, knitted polymer scaffolds coated with type I collagen can support cell attachment and proliferation and modulate mechanical properties (Sahoo et al., 2007). Zhang B. et al. (2018) also demonstrated that BMSC-collagen sponge construct combined with cyclic stretch and TGF-β1 can promote tendon healing in Achilles functional index (AFI) measurement, morphological observation, histological analysis, and mechanical testing. Despite the advantages, collagen is compromised by the drawbacks of rapid degradation kinetics and poor structural stability as well as potentially their immunogenic character due to animal origin (Citeroni et al., 2020).

Fibrin is the product of blood-derived fibrinogen and thrombin with the ability to support cell adhesion, migration, growth, and differentiation based on the structure of hydrogel or fibrous scaffolds (Hankemeier et al., 2009). As the product of patient’s own blood, fibrin can be used as an autologous scaffold with good biocompatibility and without the potential risk of foreign body reaction or infection (Ahmed et al., 2008). Fibrin is characteristic of both elastic and viscous properties, high seeding efficiency, adhesion capabilities and uniform cell distribution with the potential to bind, retain, present, and possibly release various biomolecules to modulate cell behavior (Roberts et al., 2020). The drawbacks of easy shrinkage and poor mechanical stability make fibrin cannot preserve its internal spacing and macromorphology without an external framework (Rojas-Murillo et al., 2022). Some literatures have reported the effects of composite scaffold composed of fibrin and other polymers to promote the mechanical property. Fibrin can be combined with knitted PLGA scaffolds to form a composite construct facilitating cell attachment and ingrowth and the sustained release of bFGF. The composite scaffold loaded with MSC sheets and bFGF can facilitate tendon repair by enhancing tendon-related differentiation, biomechanical strength and histological microstructures (Zhao et al., 2019). The biodegradation property is an advantage of fibrin and the process is mediated by plasmin (Park and Woo, 2018). However, the potential of fibrin hydrogel as a scaffold is compromised by the disadvantage of rapid degradation (Ahmed et al., 2008). At present there are some strategies to promote stability of fibrin hydrogel, including concomitantly optimizing pH and the concentrations of fibrinogen and calcium ion (Ca^2+^) (Eyrich et al., 2007); modifying fibrin structure with a molecule (Dikovsky et al., 2006); reducing the cell density (Passaretti et al., 2001); cross-linking (Rivkin et al., 2007); protease or plasmin inhibitors (Ahmed et al., 2007).

Silk fibroin (SF) is extracted from silkworm silk and has been used as a potential biopolymer for TE due to its excellent biocompatibility, controllable biodegradability, bioresorbability, non-cytotoxicity, low immunogenicity, non-inflammatory characteristics, haemostatic properties, and tunable mechanical properties (Kundu et al., 2013; Yao et al., 2016). Because of its outstanding mechanical properties, SF is an ideal choice for tendon tissue engineering to load tensile stress. Despite the above advantages of SF for TE, there are still some disadvantages cannot be ignored, including poor cell adherence and growth, low biodegradability (Cao and Wang, 2009). To cope with the drawbacks of not sufficient cells attach and growth, SF is often combined with other polymers to fabricate SF-based biomaterials with enhanced biological behavior (Cai et al., 2017; Sarıkaya and Gümüşderelioğlu, 2021; Yin et al., 2021). Some literatures also reported the strategies to tune the degradation rate of SF. We know that SF can be enzymatically degraded at a relative slow rate because of its special crystallization and orientation, as well as compact structure (Cao and Wang, 2009). The degradation rate of SF materials is related to the state of raw silk, parameters occurring during material fabrication processing, morphological features and host factors. There are some strategies for tuning the SF degradation rate, including manipulation of molecular weight, crystalline level, crosslinking degree, blending with another polymer, and incorporation of enzyme-sensitive peptides (Umuhoza et al., 2020). After enzymatic digestion, SF degrades into amino acids and peptides that cause no immunogenic response (Cao and Wang, 2009). In contrast, Gellynck et al. (2008) reported that silk fractions can induce mild proinflammatory cytokine release and phagocytosis. Although with some drawbacks, multiple studies have demonstrated that SF is a promising biomaterial for applications in tendon repair. Silk has shown tenogenic potential, as well as regenerative potential at the tendon level (Ruiz-Alonso et al., 2021). Sarıkaya and Gümüşderelioğlu (2021) showed that silk fibroin/poly-3-hydroxybutyrate scaffolds with aligned topography can sustain adipose-derived mesenchymal stem cell (ADSCs) proliferation and differentiation into tenocytes. Lu et al. (2020) demonstrated that an SF film with biophysical cues can support tendon stem/progenitor cell adherence, arrangement and tenogenic differentiation. Poly (p-dioxanone) (PPDO)/SF composite scaffolds with parallel and crimped fiber arrangement features showed a lower inflammatory response and could maintain cell adhesion, proliferation, and phenotypic maintenance better than pure PPDO scaffolds (Wu et al., 2021).

#### 5.1.2 Synthetic materials

Synthetic biomaterials such as the polyglycolic acid (PGA) (Day et al., 2004), polylactic acid (PLA) (Santoro et al., 2016), polylactic-co-glycolic acid (PLGA) and polycaprolactone (PCL) family of polymers (Siddiqui et al., 2018) have been used widely in tissue engineering due to their good biomechanical properties, biodegradability and commercial availability. Compared with natural material scaffolds, the synthetic polymer scaffolds showed superior mechanical properties. However, they lack biological signals and cell binding ligands and do not show optimal capability for cellular adhesion, growth, and infiltration or new tissue formation (Rinoldi et al., 2021).

##### 5.1.2.1 PGA, PLA and PLGA

Although compromised by the side effects of acidic degradation products, PGA, PLA and their copolymer PLGA are favorable polymers that are widely applied in tissue engineering because of their biocompatibility, biodegradability, good mechanical properties and excellent processability (Araque-Monrós et al., 2020; Mao et al., 2021). PLGA is the most frequently used applications of PLA and PGA in tendon tissue engineering has by the properties of excellent biocompatibility and tunable mechanical and degradation properties. The hydrophilicity of PLGA can be modulated according to the lactic and glycolic acid ratio. The degradation rate can also be controlled by changing the ratio of PLA to PGA (Mao et al., 2021). In order to avoid the toxic effects of acidic degradation products on the implanted cells and host tissues, the initial *in vitro* tendon tissue engineering followed by *in vivo* implantation may be an alternative (Cao et al., 2006). Surface modification with adhesive agents such as fibronectin can enhance cell adhesion (Qin et al., 2005). And that, Manning et al. (2013) developed a scaffold comprising electrospun nanofiber PLGA backbone and heparin/fibrin hydrogel containing platelet-derived growth factor BB (PDGF-BB), along with ADSCs, to repair tendon injury. The *in vitro* studies demonstrated that the scaffold can support cell survival and growth factor release. The *in vivo* studies demonstrated that the scaffold triggered a mild immune response and promoted tendon healing. As the simplest linear aliphatic polyester with hydrophilic property, PGA is characteristic of lack of toxicity, immunogenicity and accumulation in the cells and tissues (Song et al., 2019). The application of PGA scaffolds is limited due to their lack of functional groups for signalling molecules. The manipulation of structural parameters in the design of scaffolds and their bioactivation, through the incorporation of soluble and insoluble signals for promoting cell activities, is likely to improve the neoformation of tissues (Rodrigues et al., 2013). As a polyester of lactic acid or 2-hydroxypropionic acid, PLA is characteristic of biocompatibility, mechanical properties and hydrolytic degradation (Araque-Monrós et al., 2020). PLA is compromised by the disadvantage of hydrophobic property. Xie et al. (2022) fabricate a scaffold combining the mechanical robustness of PLA and the biological activity of collagen to promote tendon repair.

##### 5.1.2.2 PCL

PCL is a synthetic biodegradable aliphatic polyester and is characteristic of good processability, biodegradability, and biocompatibility, low melting temperature, excellent mechanical resistance, and relatively low cost (Backes et al., 2022). However, PCL has poor cellular attachment and proliferation owing to its hydrophobicity, inadequate wettability, and lack of bioactive functional groups (Lim et al., 2021). Blending PCL with natural polymers or functionalizing its surface with short stretches of amino acids and peptide sequences can enhance its biocompatibility (Siddiqui et al., 2018). It was reported that co-electrospinning of PCL/gelatin scaffolds can enhance the hydrophilicity of PCL and preserve mechanical strength to provide the regenerated tissue with good mechanical properties and histological morphology, thus restoring the function of the tendon (Yang et al., 2016; Sheng et al., 2019).

Synthetic biomaterials can share mechanical loads with the host tissue to promote the repair process by virtue of their excellent mechanical properties. Compared with their natural counterparts, synthetic biomaterials have a lower risk of disease transmission when implanted in the host body (Nau and Teuschl, 2015). Lacking biological cues in synthetic biomaterials compromises the biocompatibility The incorporation of other natural biomaterials can correct the inferior biological response of cells cultured on the scaffolds. They also have the defect of acid release in the hydrolytic biodegradation process, which can be toxic to surrounding cells. The complications of postoperative infection, chronic immune response and poor cellularity cannot yet be neglected. Furthermore, other concerns about synthetic biomaterials include the solvents used to dissolve them and the degradation products within the body. In all, we still have some research to be done to find or fabricate new polymers with the combined advantages and without disadvantages aforementioned.

### 5.2 Scaffold fabrication techniques

**FIGURE 3:**
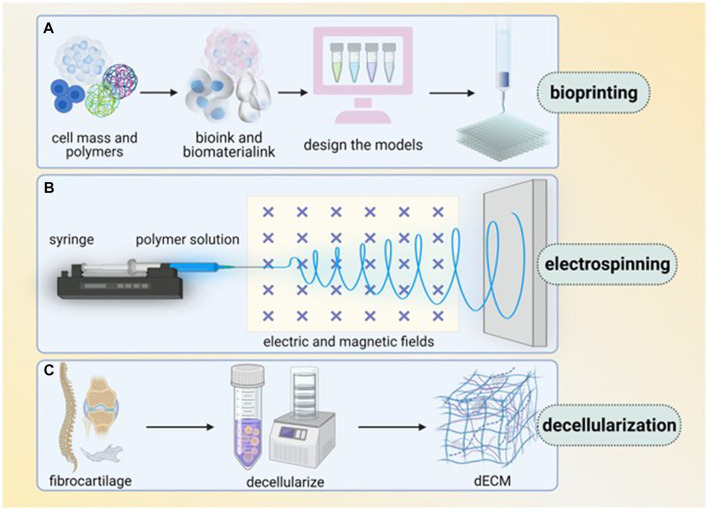
Main techniques of enthesis tissue engineering (several techniques are commonly used in enthesis engineering, including bioprinting, electrospinning and decellularization. **(A)** Bioprinting, in which bioink and biomaterial ink are extracted from cell mass and polymers, then models are designed and printed layer by layer. **(B)** Electrospinning use syringe to let polymer solution pass through electric and magnetic fields. **(C)** Decellularization transfers fibrocartilage tissue into dECM). Reproduced with permission of Oxford University Press (Li et al., 2023).

In addition to suitable biomaterials, the fabrication techniques to construct ideal scaffolds that can mimic the native tendon structure are important factors to consider. Convenient and versatile fabrication techniques are also an important issue for future large-scale clinical applications. There are many tendon tissue engineering scaffold fabrication techniques, including electrospinning, electrohydrodynamic jet printing, 3D bioprinting and freeze-drying (Figure 3).

#### 5.2.1 Electrospinning

Electrospinning is a unique approach using electrostatic forces to produce fine fibers from polymer solutions or melts. Basically, an electrospinning system consists of three major components: a high voltage power supply, a spinneret and a grounded collecting plate and utilizes a high voltage source to inject charge of a certain polarity into a polymer solution or melt, which is then accelerated towards a collector of opposite polarity (Bhardwaj and Kundu, 2010). According to the different copolymer dissolution methods, electrospinning can be classified into conventional electrospinning and melt electrospinning.

Electrospinning has been widely used in tendon tissue engineering (Guner et al., 2020) by the properties of producing ultrafine fibers resembling the nanofibrillar components found in the ECM of native tissues. Nanofiber membranes fabricated by the electrospinning technique possess a high surface-to-volume ratio and high volume of interconnected porosity, which are available for cell attachment and proliferation (Ngadiman et al., 2017). And that, scaffolds fabricated by electrospinning can reproduce the typical non-linear toe region and the biomechanical properties of tendons and ligaments (Brennan et al., 2018).

In order to produce favorable electrospun scaffolds for tendon tissue engineering, various configurations of nanofibers by different setups have been developed, including mats of nanofibers, bundles and yarns, tubes and conduits, textiles of nanofibers, multiscale hierarchical scaffolds (Sensini and Cristofolini, 2018). Electrospun mats are compromised by the drawbacks of limited mechanical properties. Co-electrospinning with two different polymers and multilayering electrospinning techniques are applied to increase the mechanical properties of the nanofibrous mats (Kidoaki et al., 2005). Furthermore, bundles and yarns of nanofifibers are the alternative electrospinning configurations to overcome the mechanical limitations of the electrospun mats. Bundles and yarns of nanofibers can be further proposed by textile technique to unite these structures (Akbari et al., 2016). The central hollows of electrospun nanofibrous conduits can be filled with nanofibers or bundles/yarns of nanofibers to form multiscale hierarchical scaffolds.

The intrinsic characteristics of electrospun scaffolds in terms of fiber topography, fiber diameter, pore size and surface chemistry together with the extrinsic ones including mechanical stimuli and scaffold degradation behavior can stimulate macrophage polarization towards an anti-inflammatory phenotype and by recruiting stem cells within the damage tissue improving hence their immunomodulatory properties (Russo et al., 2022a). Thus, many modified scaffolds based on electrospun nanofibers have been developed with enhanced functions. Almeida et al. (2019) produced yarns made of continuous and aligned electrospun nanofibrous threads based on a poly-ɛ-caprolactone/chitosan polymer blend mechanically reinforced with cellulose nanocrystals, which were coated with tropoelastin and used as representative units (fascicles) of tendons. The biomimetic scaffolds modulated stem cell fate towards tenogenic differentiation and the production of a tendon-mimetic ECM by the biophysical and biochemical cues. Cold atmospheric plasma treatment can increase the hydrophilicity of electrospun PLGA microfibers with improved cell adhesion and penetration while maintaining the biocompatibility and teno-inductive properties for ovine amniotic epithelial cells (El Khatib et al., 2020a).

Aligned nanofibers can be collected by using a dynamic mechanical collector such as a cylindrical drum (peripheral speed higher than 8 m/s) and gap collectors (Sensini and Cristofolini, 2018). The nanofiber organization and alignment can influence cell morphology and the expression of ECM molecules, which are important for the ultimate structure and mechanical function of the neotendon. Zhang et al. (2015) demonstrated that compared with random fiber scaffolds, aligned fiber scaffolds with hiPSC-MSCs had a significant effect on tendon-related gene expression *in vitro* and improved the structural and mechanical properties of tendon injury repair *in vivo*. The electrospun PLGA fleeces with highly aligned fibers can induce amniotic epithelial stem cells tenogenesis through an early epithelial-mesenchymal transition with cellular elongation along the fibers’ axis and the upregulation of mesenchymal markers, followed by upregulated tendon-related genes and Collagen Type 1 (COL1) protein expression (Russo et al., 2020). Aligned PLGA microfiber fleece with smaller fiber size (1.27 µm) can synergistically boost amniotic epithelial stem cells tenogenesis and modulate their anti-inflammatory/pro-healing paracrine signaling to promote tendon healing (El Khatib et al., 2020b).

Among the various technologies explored for healing and regenerate these tissues, electrospinning is definitely one of the most promising since it combines biomimicry and manufacturing flexibility. However, despite with these promising outcomes, electrospinning has intrinsic disadvantages, such as a small pore size (<10 µm), limited cell infiltration and complicated fabrication setups. In order to deal with the drawbacks, various nanofibrous structures base on different electrospinning setups and processes have been developed, such as mats, bundles, yarns and more complex hierarchical assemblies (Sensini and Cristofolini, 2018). The 3D scaffolds with aligned electrospun microfibers may be an promising resolution for tendon tissue engineering by providing an biomimetic microenvironment for tenodifferentiation and immunomodulation (Russo et al., 2022b), favorable topological hierarchical structure, satisfied mechanical properties (Sensini et al., 2018). For these reasons, developing electrospun multiscale hierarchical scaffolds that mimicking the structure, stiffness and strength of the native tendon is the next study direction for tendon tissue engineering (Olvera et al., 2019).

Based on the electrospinning products, many post-processing modes have been developed. Thereinto, knitting and braiding are the two most popular textile techniques and are often applied in scaffold fabrication based on fiber material for tendon tissue engineering. In knitting, yarns or threads are intertwined in a series of linked loops to form a fabric with easy production and superior mechanical properties. Braiding indicates that three or more fiber strands are intertwined to produce complex structures (Wu et al., 22018). The toe region in mechanical tests of knitted fibrous scaffolds is similar to that of the native tendon. Knitting scaffolds can be tailored to interconnected porous structures beneficial for nutrient transportation and cell infiltration. However, a controlled and homogeneous distribution of seeded cells is difficult to obtain. This problem with such scaffolds can be solved by certain techniques, including natural polymer fiber network fabrication, cell sheet cultivation or the incorporation of hydrogels to provide a microenvironment for seeded cells (Rinoldi et al., 2021). Braided scaffolds have stress–strain properties similar to those of tendons and excellent resistance against abrasion, fatigue, and catastrophic failure (Freeman et al., 2011). Braiding can be applied to fabricate flexible structures with various pore diameters, geometries and mechanical properties. Braided scaffolds present the drawbacks of limited nutrition diffusion and cell infiltration in *in vivo* tests because of their limited porosity. The application of knitted scaffolds is limited by their poor cell capture capability and thereby requires electrospun fibers or gel systems to promote cell attachment (Tamayol et al., 2013). Due to the mechanical requirements of a single fiber/yarn during the process of knitting/braiding, most natural biomaterials, except for silk fibers, are excluded by their inferior mechanical properties. The knots in the knitted scaffolds cannot remodel into the hierarchical structure of native tendon tissues. Thus, postprocessing methods, such as rolling, may be necessary to improve the structural properties of the scaffolds (Shiroud Heidari et al., 2021).

#### 5.2.2 Electrohydrodynamic jet printing (E-jetting)

Developed from electrospinning, electrohydrodynamic jet 3D printing (e-jetting) can fabricate scaffolds with customized designs by layer-by-layer fiber deposition (Wu, 2021). The 3D tendon scaffolds fabricated by the e-jetting technique present high porosity and orientated micrometer-size fibers. The 3D porous structures with varied pore sizes ranging from 60 to 200 μm produced by e-jetting contribute to cell attachment, ingrowth, metabolism, and collagen type I expression when compared to electrospun scaffolds (Wu et al., 2017a). To date, PCL is the most widely used material for e-jetting despite its low yield strain/strength (Hochleitner et al., 2018). Wu et al. (2016) developed a hybrid three-dimensional (3D) porous scaffold comprising an outer portion rolled from an electrohydrodynamic jet printed poly (e-caprolactone) (PCL) fiber mesh, and an inner portion fabricated from uniaxial stretching of a heat-sealed PCL tube exhibiting comparable mechanical properties to those of the human patellar tendon. The cultured human tenocytes on the scaffolds showed upregulated cell alignment, cell elongation and formation of collagen type I. In another study, Wu et al. (2017b) further revealed that the 3D tendon scaffold exhibited the effects of tendious gene expression upregulation and the consistency between the weight loss and the decline of mechanical properties.

Despite with the advantages, there are some limitations that cannot be ignored. The e-jetting technique cannot fabricate scaffolds with thicknesses greater than 7 mm due to the semiconductive property of the solution or polymer melt (Wunner et al., 2018). In addition, the interfiber distances cannot be too small because of the electric field interference of neighboring fibers (Wu, 2021). And that, the hydrophobicity of the applied biomaterials (e.g., PCL) in e-jetting affects the efficiency of cell seeding on them. In order to improve hydrophobicity, surface modification can promote cell adhesion on these scaffolds, including treatment with alkaline hydrolysis (Wu et al., 2017a) or coating with collagen (Cai et al., 2013).

#### 5.2.3 Wet electrospinning

Replacing a classical metal collector with a liquid bath to collect nanofifibers is the operating principle of the so-called wet electrospinning (wet-spinning) (Sensini and Cristofolini, 2018). The wet-spinning technique can fabricate fibers with random or aligned orientations by ejecting a polymer solution though a syringe into a liquid crosslinking bath (Yang et al., 2017). Wet spinning can convert natural polymers, such as gelatin and collagen, into aligned biomaterial fibers with good appearance and favorable characteristics. Wang et al. (2019a) utilized wet spinning to construct gelatin-based filaments and tube structures with excellent biocompatibility and cell environments that can be used in prospective tissue engineering applications. Nowotny et al. (2016) processed chitosan fibers to a novel pure high-grade multifilament yarn with reproducible quality based on wet spinning technology and then braided the fibres into a 3D tendon scaffold. The fabricated CS scaffolds demonstrate similar tensile properties as human tendons and allow a good adhesion and proliferation of human mesenchymal stem cells.

An advantage of wet spinning is the absence of toxic solvent during the spinning process, which is beneficial for cell attachment and proliferation. And that, compared with the electrospinning method, the scaffolds fabricated by wet spinning exhibit a higher porosity and larger pore size, facilitating cell adhesion, proliferation, and penetration (Gurel Pekozer and Torun Kose, 2019). Wet-spinning does not use high voltages or temperatures reducing the denaturation possibility of the sample (Ruiz-Alonso et al., 2021). In addition to all this, once produced, the fibers are easily handled and assemble, making wet-spinning a low cost and high yield technique (Calejo et al., 2019). However, the inferior mechanical properties of wet spinning scaffolds hinder their application in tendon tissue engineering. The composite silk/collagen scaffolds fabricated by wet spinning exhibited greater initial ultimate tensile stress than the human ACL and met the mechanical requirements for ACL construction (Panas-Perez et al., 2013).

#### 5.2.4 Other seldom used fabrication techniques

**FIGURE 4:**
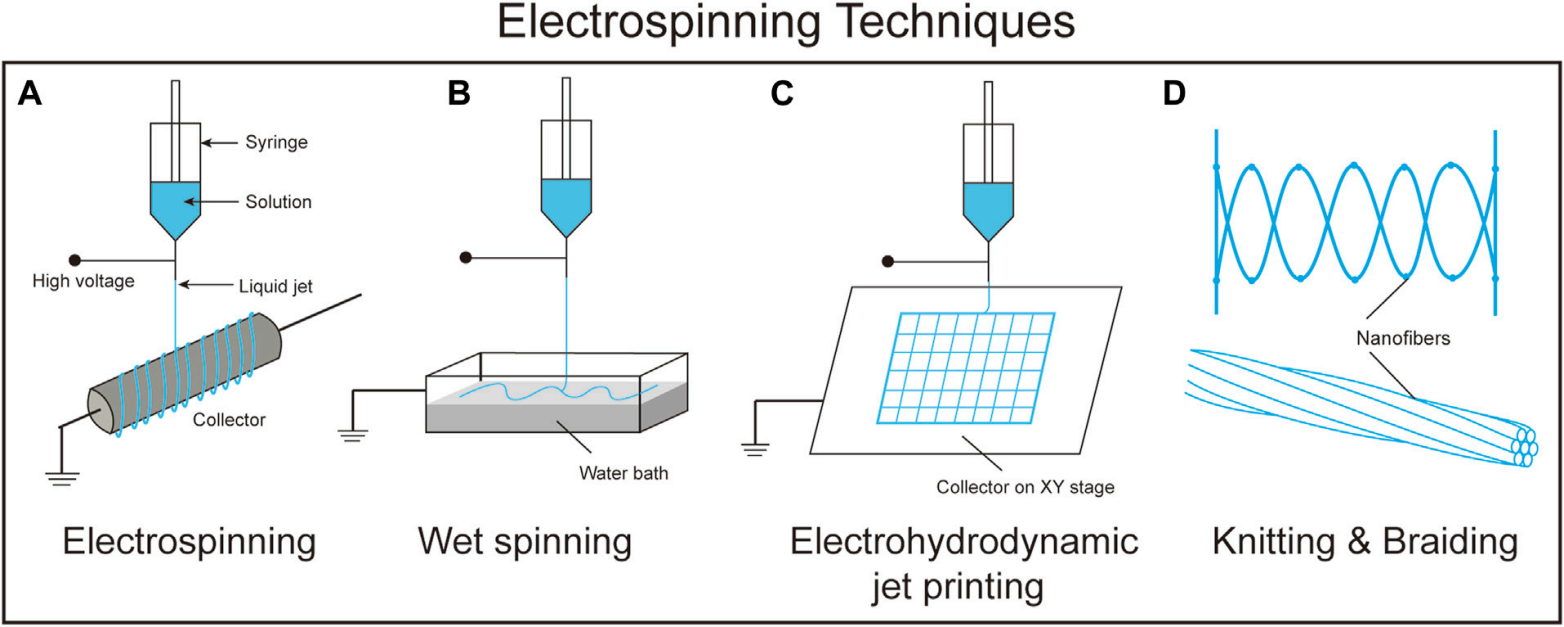
A schematic comparison between the different electrospinning techniques. **(A)**. Electrospinning, **(B)**. Wet spinning, **(C)**. Electrohydrodynamic jet printing, and **(D)**. Knitting and braiding.

The technique of 3D bioprinting can provide viable, autologous tissue-like constructs to repair tissue defects (Potyondy et al., 2021). Extrusion-based, inkjet-based and light-based bioprinting are the three main modalities of 3D printing. For 3D bioprinting, the properties of biomaterials and the printing parameters are the basis to support favorable geometric accuracy and cell viability. Although 3D bioprinting is a promising technique in tissue engineering, the absence of the capability to mimic the function of native tendon is a shortcoming. Balancing the bioactivity and mechanical properties of biomaterials used as bioinks remains a challenge. Combining natural inks with more biocompatible materials, such as PLGA, PLA, and PCL, can resolve this problem. Mozdzen et al. (2016) incorporated 3D bioprinting acrylonitrile butadiene styrene fibers into a collagen scaffold to form a composite scaffold with controllable mechanical performance. Further research should be focused on exploring materials and printing techniques that can satisfy the requirements of mechanical strength and the cell proliferation microenvironment for tendon tissue engineering (Potyondy et al., 2021) (Figure 4).

The freeze-drying technique has been used in tendon tissue engineering to form scaffolds with tailored porosity that can facilitate cell migration, infiltration and proliferation. Natural biopolymers, such as collagen and silk, are often applied in freeze-drying techniques due to their favorable biocompatibility and can promote ECM formation in cooperation with the porous structure of the scaffold (Sun et al., 2021). Zheng et al. (2016) fabricated two different orientation scaffolds by the freeze-drying techniques and presented that parallel collagen scaffold can induce tendon stem cells to be in spindly and parallel arrangement, and promote parallel extracellular matrix formation; while random collagen scaffold can induce cells in random arrangement. The results indicate that parallel collagen scaffold is an ideal structure to promote tendon repairing. However, the poor mechanical properties of freeze-dried scaffolds are the main hindrance for tendon tissue engineering. Combination with other polymers to form composite scaffolds with enhanced mechanical properties is a feasible solution (Sandri et al., 2016).

Although some progress in scaffold fabrication techniques has been made in tendon tissue engineering, we still face obstacles to overcome for further clinical application. Biologically stable elements based on scaffolds are beneficial for tendon tissue engineering to promote the repair process. The current polymers for scaffold fabrication are limited by adverse processing (dissolution in toxic solvents or exposure to high temperature) for integration with bioactive reagents. Thus, how to enable scaffolds to deliver bioactive reagents to enhance tissue repair needs further study. Different fabrication techniques have their own advantages and disadvantages, so the combination of two or more methods to enhance the structure and biofunction merits of the scaffold is desirable.

## 6 Cells for tendon tissue engineering

As one component of the tissue engineering triad, cells are often combined with scaffolds to facilitate tissue repair and regeneration. To date, there are two main cell types used in tendon tissue engineering, i.e., stem cells and differentiated cells. The commonly used stem cells in tendon tissue engineering include MSCs and pluripotent stem cells (PSCs), which exhibit the properties of self-renewal and multilineage differentiation. Tenocytes and fibroblasts are the main differentiated cells in tendon tissue engineering. The advantages and disadvantages of the commonly used cells for tendon tissue engineering are summarized in Table 2.

**TABLE 2:** Summary of the main advantages and disadvantages of the cells used for tendon tissue engineering.

Cell type	Cell name	Advantages	Disadvantages	References
Mesenchymal stem cells (MSCs)	Adipose-derived mesenchymal stem cells (ASCs)	Easy availability, minimal harm, abundant cell numbers, simple expansion steps *in vitro*, multilineage differentiation potential	Risk of differentiation into unwanted cell lineages	Backes et al. (2022), Yang et al. (2016), Zhang Q. et al. (2021), Zuk et al. (2001), Chen S. et al. (2021), Park et al. (2010), Chen S. H. et al. (2021), Lee et al. (2017), Costa-Almeida et al. (2018)
	Bone marrow mesenchymal stem cells (BMSCs)	Easily harvested, high proliferation rate and tendon-related mRNA, protein expression levels, low immunogenicity	Complications of ectopic bone and tumor formation, phenotypic drift, painful harvesting procedures, low cell yield	Bi et al. (2007), Lui et al. (2011), Baberg et al. (2019), Citeroni et al. (2020)
	Tendon stem/progenitor cells (TSPCs)	Tendon-derived cell type with inherent pro-tenogenic abilities, increased collagen production and fiber alignment, improved cell alignment, enhanced mechanical properties	No specific molecular markers, donor site morbidity, poor obtainable number, phenotypic drift during *in vitro* expansion	Schulze-Tanzil et al. (2004), Bi et al. (2007), Rui et al. (2010), Lui and Chan (2011), Dex et al. (2016), Komatsu et al. (2016), Laranjeira et al. (2017), Zhang C. et al. (2018), Schneider et al. (2018), Migliorini et al. (2020)
Pluripotent stem cells	Embryonic stem cells (ESCs)	Excellent proliferative capability and differentiation capacity	Ethical controversy, risk of teratoma or ectopic bone formation, risk of differentiation into unwanted cell lineages	Crook et al. (2007), Yin et al. (2010), Lim et al. (2019)
	Induced pluripotent stem cells (iPSCs)	Excellent proliferative capability and differentiation capacity	Risk of teratoma formation, risk of differentiation into unwanted cell lineages	Jung et al. (2012); Harding and Mirochnitchenko (2014); Zhang et al. (2015)
Amnion-derived stem cells	Amniotic epithelial cells (AECs)	Renewal, multi-differentiation potential, no-tumorigenicity, low/no immunogenicity, no ethical or legal concerns, paracrine effects potent, immunomodulatory effects	Unstable characterization; cell quality, safety and efficacy concern	Antoniadou and David (2016), Abbasi-Kangevari et al. (2019), Liu Q. et al. (2021)
	Amniotic mesenchymal stromal cells (AMSCs)			
Differentiated cells	Tenocytes	Natural tendon cell population, no risk of teratoma	Phenotype drift and functional loss during *in vitro* expansion, low proliferation potential, difficult to harvest, risk of injury of the donor tissue	Lui et al. (2011), Mazzocca et al. (2012), Lui, (2015), Kirk et al. (2021), Ruiz-Alonso et al. (2021)
	Fibroblasts	Can differentiate into tenogenic linages, easy to obtain with minimal injury to the donor site, easily expanded during *in vitro* culture, no risk of teratoma	Terminally differentiated cell type with a limited lifespan	Liu et al. (2006); Wang (2006); Yin et al. (2010); Mazzocca et al. (2012)

### 6.1 Stem cells for tendon tissue engineering

Stem cells have the following advantages: the ability to proliferate and synthesize active paracrine factors, the ability to promote immune regulation and thus tendon regeneration, and the potential to differentiate into tendon cells. Thus, stem cells have great application value in tissue engineering for tendon repair (Lim et al., 2018; Schneider et al., 2018).

#### 6.1.1 Mesenchymal stem cells (MSCs)

MSCs can be isolated from various tissues, including adipose tissue, bone marrow, synovium, periosteum, articular cartilage, and umbilical cord. MSCs can differentiate into tendon cells, synthesize ECM, secrete paracrine factors, and modulate inflammation to promote tendon regeneration.

Adipose-derived mesenchymal stem cells (ADSCs) are characterized by easy availability, minimal harm, abundant cell numbers and simple expansion steps *in vitro*. They exhibit multilineage differentiation potential toward adipogenic, osteogenic, chondrogenic, and myogenic lineages *in vitro* when cultured with lineage-specific differentiation factors (Zuk et al., 2001). The aforementioned merits make ADSCs a popular cell source for tendon tissue engineering (Chen J. et al., 2021; Sarıkaya and Gümüşderelioğlu, 2021). ADSCs exhibited much greater potential for the adipogenic lineage. However, coculture with growth differentiation factor-5 can induce the tenogenic differentiation of ADSCs and enhance ECM (collagen type I, decorin, and aggrecan) and tendonogenic marker (scleraxis, tenomodulin, and tenascin-C) gene expression (Park et al., 2010). Tendon defects treated with hADSCs showed better gross morphological and biomechanical properties and expressed more collagen type I and tenogenic genes tenascin-C than the defects in the fibrin and sham groups (Lee et al., 2017). Achilles tendon defects in rabbits treated with ASC-mixed platelet-rich plasma (PRP) expressed more collagen type I, FGF and VEGF and lower transforming growth factors-β1, 2, 3 compared with PRP gel groups. The mechanisms of ADSCs improve tendon healing may be attributed to cellular crosstalk between ADSCs and tendon cells with an upregulation of tendon-related genes (Costa-Almeida et al., 2018). And that, ADSCs may have a beneficial effect toward shifting the tendon repair microenvironment. The high proliferation rate of ADSCs is not consistent with the biological properties of low cell numbers in tendon, which may lead to fibrotic tissue formation and scarring (Costa-Almeida and Gomes, 2019). Differentiation into adipogenesis is the main disadvantage of ADSCs (Neo et al., 2016).

Bone marrow mesenchymal stem cells (BMSCs) can be easily harvested from the iliac crest or long bones *via* bone marrow aspiration and are the most widely used stem cell type. BMSCs express several tendon-related markers, including collagen types I, II and III, scleraxis, tenomodulin, decorin and biglycan, although to a lower extent than TSPCs (Bi et al., 2007). The crosstalk between tendon cells and BM-MSCs with increased expression of tendon-related genes and tendon ECM markers during *in vitro* co-culture process has been demonstrated. BM-MSCs can differentiate into tenocytes by exposure to growth factors such as GDF5 and BMP14 and/or mechanical stimulation (Citeroni et al., 2020). Multiple growth factors, such as BMP-2 and BMP-12, and active Smad8 can also induce the differentiation of BM-MSCs into the tenogenic lineage (Schneider et al., 2018). The low immunogenicity of BM-MSCs makes allogenic transplantation possible. BM-MSCs can positively modulate inflammatory reactions to facilitate tendon repair. Furthermore, BM-MSCs can support tendon cells by paracrine role with releasing several growth factors, cytokines and other soluble molecules (Baberg et al., 2019). Despite these encouraging findings, wide application is hindered by the complications of ectopic bone and tumor formation (Lui et al., 2011). BM-MSCs also face the problems of phenotypic drift, painful harvesting procedures with frequently low cell yield, and reduced BMSC quality with advanced donor age (Lui et al., 2011).

Tendon stem/progenitor cells (TSPCs) were first identified in human and mouse tendons by Bi et al. (2007) and then confirmed in various tendons and ligaments from different species (Schneider et al., 2018). TSPCs are multipotent adult stem cells with the properties of clonogenicity, multilineage differentiation potential and specific stemness surface marks (Migliorini et al., 2020). As resident cells in tendons, TSPCs account for approximately 4% of the tendon cellular population and may be a safer and better alternative to other types of stem cells (Bi et al., 2007). TSPCs are a tendon-derived cell type with inherent proteinogenic abilities. They have been used alone or in combination with other substances in the field of tendon regeneration tissue engineering (Komatsu et al., 2016). TSPCs express some stem cell markers, such as CD44, CD90, CD91, CD105, CD146, Oct-4 and SSEA-1 (Lui and Chan, 2011), and the tendon-related genes scleraxis (Scx), tenomodulin (Tnmd) (Dex et al., 2016), cartilage oligomeric matrix protein (Comp) and TNC (Rui et al., 2010). Since there are no specific molecular markers to discriminate TSPCs from tenoblasts and tenocytes, it is impossible to isolate pure subsets of cell populations from these differentiation stages (Schneider et al., 2018). Donor site morbidity, poor obtainable number and phenotypic drift during *in vitro* expansion limit the application of TSPCs. Three-dimensional (3-D) pellets mimicking the natural niche can prevent cell phenotype loss and preserve the expression of the tendon-related genes Scx, Col I and Col II over 14 days (Schulze-Tanzil et al., 2004). TSPCs can also sustain stable cell phenotypes when they are cultivated on 3-D scaffolds mimicking native tendon ECM molecular composition, organization, topography or are subjected to mechanical stimulation (Laranjeira et al., 2017). Epigenomic approaches to maintain the tenogenic phenotype of TSPCs is the recent developed technology (Zhang Y. J. et al., 2018). TSPC implantation in tendon defects can significantly increase tendon healing with increased collagen production and fiber alignment, improved cell alignment, and enhanced mechanical properties (Ni et al., 2012). Although some progresses have been made to facilitate TSPCs toward clinical application, we still need to reduce additional steps of cellular manipulation *in vitro*.

Compared to PSCs, MSCs do not increase the risk of developing teratoma and are easier to obtain without ethical concern. However, there are still some defects that need to be solved, such as standard MSC isolation, expansion, culture conditions, optimal cell-seeding density, control of the differentiation to tendon and reduction in ectopic bone formation (Ouyang et al., 2006). To circumvent the problems of ectopic bone and tumor formation, the *in vitro* induction of MSCs into tendon progenitors before transplantation is a possible alternative to promote tendon repair (Lui et al., 2011). And that, despite current studies focusing on tenogenic differentiation of MSCs, the therapeutic effects may be exerted by the empowering capacity of stem cells over tendon resident cell populations (Costa-Almeida and Gomes, 2019). A deeper understanding of molecular mechanisms as well as signaling pathways will help identify the best cell type in tissue engineering. The functional mechanism and final destination of MSCs and their interactions with other cells need to be further researched.

#### 6.1.2 Pluripotent stem cells (PSCs)

The two main categories of PSCs, embryonic stem cells (ESCs) and Induced pluripotent stem cells (iPSCs), have great proliferative capability and differentiation capacity. ESCs have the ability to differentiate into all tissues derived from the three germ layers and proliferate (Crook et al., 2007). ESCs are characteristic of greater survival and migration capacities throughout the tendon compared to MSCs and the potential of providing sufficient cell numbers by their incomparable proliferation capacity. However, the application of ESCs may increase the risk of teratoma or ectopic bone formation after administration and may raise ethical concerns caused by harmful acquisition from human embryos (Lim et al., 2019). Purifying the relevant cell types from ESCs *in vitro* is a necessary step to avoid teratoma formation. Manipulating the culture conditions to control the ESCs differentiating into functional tenocytes is beneficial for tendon regeneration (Yin et al., 2010). Due to the origin from differentiated cells, ethical concerns about iPSCs may be avoided. However, as the pluripotent stem cells, teratoma formation during proliferation and differentiation remains unsolved (Harding and Mirochnitchenko, 2014). Removing undifferentiated iPSCs by induction and isolation is a viable measure with improved biosafety compared with the direct use of iPSCs (Jung et al., 2012). Some studies have reported favorable results about the induction approaches and therapeutic effects. Nakajima et al. (2021) derived xeno-free tenocytes from human iPSCs by recapitulating the normal progression of step-wise narrowing fate decisions in vertebrate embryos and demonstrated that iPSC-tenocyte grafting contributed to motor function recovery after Achilles tendon injury in rats *via* engraftment and paracrine effects. Zhang et al. (2015) developed a robust stepwise differentiation strategy for teno-lineage differentiation by sequentially changing physical substrate properties rather than directly differentiating iPSCs into tenocytes. By the properties of high proliferative ability, relatively low risk of ectopic tissue formation, without ethical concerns and autologous tissue harvest procedures, iPSCs derived tenocytes may represent a promising alternative for tendon injury repair.

In conclusion, PSCs may be used as a vital cell type for tendon repair. The successful induction of tenocytes from human iPSCs is a critical procedure for the further safe utilization. Elucidating the developmental mechanism of tenocytes based on development-informed protocols can provide us with new perspective to resolve the induction problem (Nakajima and Ikeya, 2021).

#### 6.1.3 Amniotic mesenchymal stromal cells (AMSCs)

Except for mesenchymal stem cells and embryonic stem cells mentioned above, placenta is now also recognized as an alternative plentiful source of multipotent stem cells. Placental stem cells possess the favorable biological properties for regenerative medicine applications such as renewal, multi-differentiation potential, no-tumorigenicity, low/no immunogenicity, no ethical or legal concerns and their potent paracrine effects, especially immunomodulatory effects, making them an promising therapeutic cell type available (Antoniadou and David, 2016). The placental cells can be divided into the amniotic epithelial cells (AECs), Amniotic mesenchymal stromal cells (AMSCs), chorionic mesenchymal stromal cells (CMSCs) and chorionic trophoblast cells (hCTCs). Thereinto, AESCs and AMSCs compose amnion-derived stem cells (Liu Q. W. et al., 2021). AECs are derived from the amnion membrane by differential enzymatic digestion methods (Barbati et al., 2012) and AMSCs are derived from the extraembryonic mesoderm.

AMSCs can modulate and suppress inflammatory responses by reducing activities of inflammatory cells and inhibiting migration of microglia and recruitment of immune cells to injury sites (Abbasi-Kangevari et al., 2019). AMSCs also exhibited angiogenic, cytoprotective, anti-scarring, and antibacterial properties (Carvajal et al., 2016). Based on the properties, we may prudently conclude that AMSCs may be a potential cell source for cell-based therapy of diseases. AMSCs have been applied for gynecological diseases, nervous system, lung, liver, cancer, skin, bone, cardiovascular and muscular skeletal diseases and so on. The paracrine effects of AMSCs may be the underlying mechanism of therapeutic efficacy. It has been demonstrated that equine amniotic microvesicles are able to target tendon cells and exert anti-inflammatory effect (Lange-Consiglio et al., 2016).

Several studies have explored the possibility of AMSCs-based therapy for tendon injury. Lange-Consiglio et al. (2013a) investigated the efficacy of AMSCs compared with bone marrowe-derived mesenchymal stromal cells (BM-MSCs) in equine tendon and ligament injuries, and concluded that AMSCs presented higher plasticity and proliferative capacity than BM-MSCs demonstrated by the shorter rehabilitation time and the lower re-injury rate of horses in AMSCs group. Except direct application, AMSCs can also be combine with other factors and cytokines to promote repair or reconstruction of an injured tendon structure. AMSCs modifed with Scleraxis (Scx) enhanced tenogenic differentiation and tendon-like tissue formation (Zhu et al., 2020).

Although with some positive effects, some problems limiting the application of AMSCs cannot be ignored. The characterization of AMSCs can be affected by the gestational age, region of cell isolation in placenta, isolation protocols, cross-contamination, passage numbers, and measuring methods, *etc.* Therefore, how to keep the characterization of AMSCs in a stable state is necessary for the further application. Besides, standard manufacturing and cryopreservation process should be established to guarantee the cells quality. How to avoid acute adverse events including local site reaction, anaphylaxis, infection, features of rejection, and tumor formation to ensure safety application should also be concerned (Liu Q. et al., 2021).

### 6.2 Differentiated cells

Tenocytes and fibroblasts are the two main unipotent cell types. They have the advantage over stem cells that they do not produce teratomas (Kirk et al., 2021). They can expand *in vitro* with a limited expansion capacity, but lack self-renewal capability *in vivo*. Deficient functions caused by phenotype drift with increasing passaging is also a concern (Mazzocca et al., 2012).

As the main cell type in tendons, tenocytes can synthesize ECM and produce growth factors to enhance tendon repair (Lui, 2015). The other advantage of Tenocytes is that they do not produce teratomas (Ruiz-Alonso et al., 2021). The first limitation of tenocytes is phenotype drift and functional loss combined with decreased expression of tendon-specific genes, such as decorin, tenomodulin (Tnmd) and thrombospondin 4 (Thbs4), during *in vitro* expansion. The second limitation is the low number of tenocytes in the normal tendon and the low proliferation potential in the *in vitro* culture process (Lui et al., 2011). How to support a stable phenotype and promote proliferation capacity of tenocytes is a vital issue to resolve. The harmful acquisition from normal tendon is also a problem that cannot be avoided.

Fibroblasts share more common characteristics with tenocytes and devoid of the disadvantage of donor site morbidity caused by tenocytes acquisition. Fibroblasts are the major cell population aligned in rows between collagen fiber bundles in connective tissue and are critical to maintaining structural integrity by synthesizing ECM proteins (e.g., collagens, fibronectin, and proteoglycans) and by producing and remodeling the collagen matrix (Wang, 2006). More recently, they have been recognized as a highly functionally diverse cell population that constantly responds and adapts to the environment by the property of biological memory (Kirk et al., 2021). Fibroblasts can be harvested from a small piece of skin tissue easily without major donor site defect (Liu et al., 2006). Furthermore, fibroblasts do not produce teratomas, which is an advantage over stem cells (Ruiz-Alonso et al., 2021). And that, fibroblasts are easily expanded during *in vitro* culture. However, fibroblasts have the inherent defect of terminally differentiated cell type with a limited lifespan, which further limits extensive clinical application (Yin et al., 2010).

## 7 Biological adjuncts for tendon tissue engineering

### 7.1 Growth factors

Growth factors are a group of cytokines that are involved in the process of cell growth, proliferation and differentiation. They participate in cell recruitment and in the stimulation of ECM synthesis, which are important for the natural regeneration of tendons (Titan et al., 2019). Commonly used growth factors include connective tissue growth factor (CTGF), insulin-like growth factor 1 (IGF-1), platelet-derived growth factor (PDGF), transforming growth factor-β (TGF-β), vascular endothelial growth factor (VEGF), basic fibroblast growth factor (bFGF), and bone morphogenetic proteins (BMPs), among others. They can be used independently or in combination with other kinds of therapies (Younesi et al., 2017). The advantages and disadvantages of the commonly used growth factors for tendon tissue engineering are summarized in Table 3.

**TABLE 3:** Summary of the main growth factors for tendon tissue engineering.

Growth factor	Main effects	References
CTGF	Promote fibroblast proliferation and matrix formation, increased expression of tenogenic genes, increased anti-inflammatory gene expression and collagen synthesis	Wang et al. (2003), Lee et al. (2006), Liu et al. (2015), Zhang B. et al. (2018), Shen et al. (2018), Li et al. (2019)
IGF -I	Promote tendon fibroblasts proliferation and migration, increase collagen and proteoglycan production	Schnabel et al. (2009), Liu et al. (2014), Holladay et al. (2016)
PDGF	Stimulate other growth factors production, increase mitogenic responses and DNA synthesis, facilitate cells migration and proliferation, increase tenogenic differentiation, upregulate Scx, Tnmd and TNC expression	Wang (2006), Branford et al. (2014), Li et al. (2021)
TGF -β	Induce extrinsic cell migration and proliferation, stimulate collagen production, regulate proteinases, initiate inflammatory response, enhance neovascularization, increase extracellular matrix deposition, promote tissue fibrosis and scarring	Chang et al. (1997), Wang (2006), Ito et al. (2010); Yin et al. (2010), Jones et al. (2013); Juneja et al. (2013), Zhou et al. (2013), Branford et al. (2014), Jiang et al. (2016), Zhang C. et al. (2018), Schneider et al. (2018), Titan et al. (2019)
VEGF	Stimulate endothelial cell proliferation, promote angiogenesis and capillary permeability, enhance collagen synthesis	Bidder et al. (2000), Pufe et al. (2005), Wang (2006), Kraus et al. (2018), Li et al. (2021)
bFGF	Involved in inflammatory response, angiogenesis, cell proliferation and collagen synthesis, promote BMSC proliferation, induce tendon-like fibroblastic differentiation, promote tendon-related mRNA expression	Sahoo et al. (2010), Tang et al. (2016)
Bone morphogenetic proteins (BMPs)	Involved in chemotaxis, proliferation, matrix synthesis and tenogenic differentiation of MSCs, modulate collagen formation	Chhabra et al. (2003), Bolt et al. (2007), Lamplot et al. (2014), Chamberlain et al. (2015), Citeroni et al. (2020)

CTGF, a downstream mediator of TGF-β, is involved in the process of tendon development and repair. CTGF can promote fibroblast proliferation and matrix formation *in vitro* (Wang et al., 2003). It was reported that the fibroblastic effect of CTGF on human BM-MSCs can increase the expression of collagen type I, tenacin-C proteins,fibroblast-related genes (Lee et al., 2006) and proteins such as Scx, Tnmd, and Col Ia. CTGF can induce the tenogenic differentiation of ADSCs *via* the FAK and ERK1/2 pathways (Li et al., 2019). CTGF can also enhance the ability of BMP12 to induce the tenogenesis of tendon stem cells *via* the Smad1/5/8 pathway (Liu et al., 2015). For intrasynovial tendon injuries, CTGF, either alone or with ADSCs, reduced inflammatory (IL1B and IL6) and matrix degrading (MMP3 and MMP13) gene expression while increasing anti-inflammatory gene (IL4) expression and collagen synthesis (Shen et al., 2018). Till now, the mechanism of CTGF regulated tenogenic differentiation and the links to other gnrowth factors signaling especially to TGF-β family signaling pathways remains unclear. In order to get an in-depth understanding of its roles in tendon regeneration, further investigation of CTGF and its potential crosstalk with TGF-β is necessary (Zhang Y. J. et al., 2018).

It was reported that IGF-I can promote the proliferation and migration of tendon fibroblasts and subsequently increase collagen and proteoglycan production to accelerate functional and structural recovery of injured tendons (Wang, 2006). However, the *in vitro* studies get non-homogeneous conclusion on the tenogenic role of IGF-1 and *in vivo* evidence collected to date did not demonstrate any tenogenic role of IGF-1 (Schnabel et al., 2009; Liu et al., 2014; Holladay et al., 2016).

PDGF is a dimeric glycoprotein composed of two identical chains (AA or BB) or a combination of two different chains (Branford et al., 2014). PDGF can stimulate the production of other growth factors and increase mitogenic responses and DNA synthesis (Wang, 2006). PDGF can also facilitate the migration and proliferation of fibroblasts, tenocytes and mesenchymal stem cells related to tissue homeostasis. PDGF increases the tenogenic differentiation of ADSCs *in vitro*, upregulating the expression of Scx, Tnmd and TNC (Schneider et al., 2018). PDGF can enhance tendon healing by promoting morphological and biomechanical properties (Li et al., 2021).

TGF-β is a protein family consisting of three isoforms (TGF-β1, TGF-β2, TGF-β3) that participate in many cellular processes intrinsic to wound healing (Branford et al., 2014). TGF-β induces extrinsic cell migration and proliferation, stimulates collagen production and regulates proteinases. TGF-β is engaged in a series of reactions, including initiating the inflammatory response, enhancing neovascularization, increasing extracellular matrix deposition and promoting tissue fibrosis and scarring (Wang, 2006; Titan et al., 2019). TGF-β plays an important role in the healing process by producing and organizing collagen fibers. Mechanical loading can induce TGF-β expression, and the TGF-β signaling pathway regulates ECM and protease expression in tendons. On the other hand, the upregulated expression of TGF-β in tendinopathy implicates its role as an indicator in tendon disorders (Jones et al., 2013). TGF-β1 is expressed by inflammatory cells, infiltrating fibroblasts, and tenocytes and is associated with scar formation. Furthermore, TGF-β1 can enhance collagen and proteoglycan synthesis, promoting injured tendon repair (Yin et al., 2010). Blocking the TGF-β1 signaling pathway can simultaneously downregulate scar tissue formation and decrease the mechanical strength of tendons (Chang et al., 1997; Zhou et al., 2013). The expression of TGF-β1 reaches a peak at the early stage and TGF-β3 at the late stage in the process of flexor tendon repair in mice (Juneja et al., 2013). Prolonged TGF-β1 stimulation may reduce ECM remodeling and lead to scar formation (Zhang B. et al., 2018). On the contrary, TGF-β3 can decreased extrinsic scarring and tendon adhesion, promote tendon healing by regulate Smad3 and Smad7 proteins (Jiang et al., 2016). TGF-β is involved in the process of tendon development. The TGFβ-Scx pathway plays a critical role in the initial differentiation of tendons and the TGFβ-Mkx pathway is essential for tendon maturation (Ito et al., 2010). The disruption of any part of the TGF-β signaling pathway will result in tendon loss. TGF-β can induce BM-MSCs and ESCs to differentiate toward the tenogenic lineage. It has been demonstrated that TGF-β1 can enhance tenogenic marker expression of MSCs. TGF-β2 isoform can induce tenogenic Col I and Scx expression. The implantation of BM-MSCs treated with TGF-β can increase collagen production and the mechanical strength of repaired tendons. In the next step, we should further study the differences in molecular mechanisms and signaling pathways among the three isoforms and the possible negative effects of TGF-β (Schneider et al., 2018).

VEGF can stimulate endothelial cell proliferation and promote angiogenesis and capillary permeability (Wang, 2006). VEGF levels increase during the stage of tendon development and decrease to a steady state in the mature stage. VEGF levels also increase during tendon healing process and tendinopathy, and decrease to normal levels in healthy tendon (Bidder et al., 2000). VEGF is associated with the differentiation of vascular and non-vascular regions in the tendon (Pufe et al., 2005). In addition to playing an important role in angiogenesis, VEGF cannot enhance the synthesis of collagen during the repair process of injured tendons. However, ectopic VEGF delivery can improve the tensile strength of injured Achilles tendon (Li et al., 2021). VEGF can promote tenocyte proliferation and increase Scx and Col I expression by downregulating Col III (Kraus et al., 2018).

bFGF is a member of the heparin-binding growth factor family and is involved in the inflammatory response, angiogenesis, cell proliferation and collagen synthesis during the tendon healing process (Tang et al., 2016). bFGF can promote BMSC proliferation, induce tendon-like fibroblastic differentiation, and promote the tendon-related mRNA expression of collagen type I, collagen type III, fibronectin, α-smooth muscle actin, and biglycan (Sahoo et al., 2010). The bFGF effect was strictly dose-dependent *in vitro* independent of stem cell source.

BMPs are a family of highly related molecules within the TGF-β superfamily. BMP-12, -13 and -14 (also known as GDF-7, -6 and -5) play important roles in chemotaxis, proliferation, matrix synthesis and tenogenic differentiation of MSCs. BMPs are elevated at early stages and decrease gradually over time during tendon healing (Citeroni et al., 2020). BMP-12 can modulate collagen formation and tendon remodeling to facilitate tendon healing. BMP13 was reported to be the most tenogenic and least osteogenic member of the BMP group. Adenovirus-mediated BMP13 in tenocytes can promote tendon healing with improved biomechanical and histologic properties (Lamplot et al., 2014). BMP-14 plays an important role in early tendon healing. BMP-14 is responsible for the establishment and maintenance of tendon properties. Knockout mice lacking BMP-14 showed alterations in collagen I arrangement and mechanical properties, resulting in delayed tendon healing (Chhabra et al., 2003). The injection of recombinant protein into tendons can induce ectopic bone or cartilage formation, which has been a concern regarding the use of BMPs to promote tendon healing. Adenovirus-mediated gene therapy can prevent the adverse effects of ectopic bone or cartilage formation (Lou, 2000).

Although some progress has been made in growth factor application, there are still some problems to be solved, such as the timing of injection, the specific dosage, and the duration of growth factors in the damaged tissue (Chan et al., 2006). Systems that can release growth factors in a gradual and controlled manner to the injury site would be an advisable alternative. Gene transfer techniques can produce growth factors in the injury site by expressing related genes continuously to promote repair (Dai et al., 2003).

### 7.2 Exosomes

**FIGURE 5:**
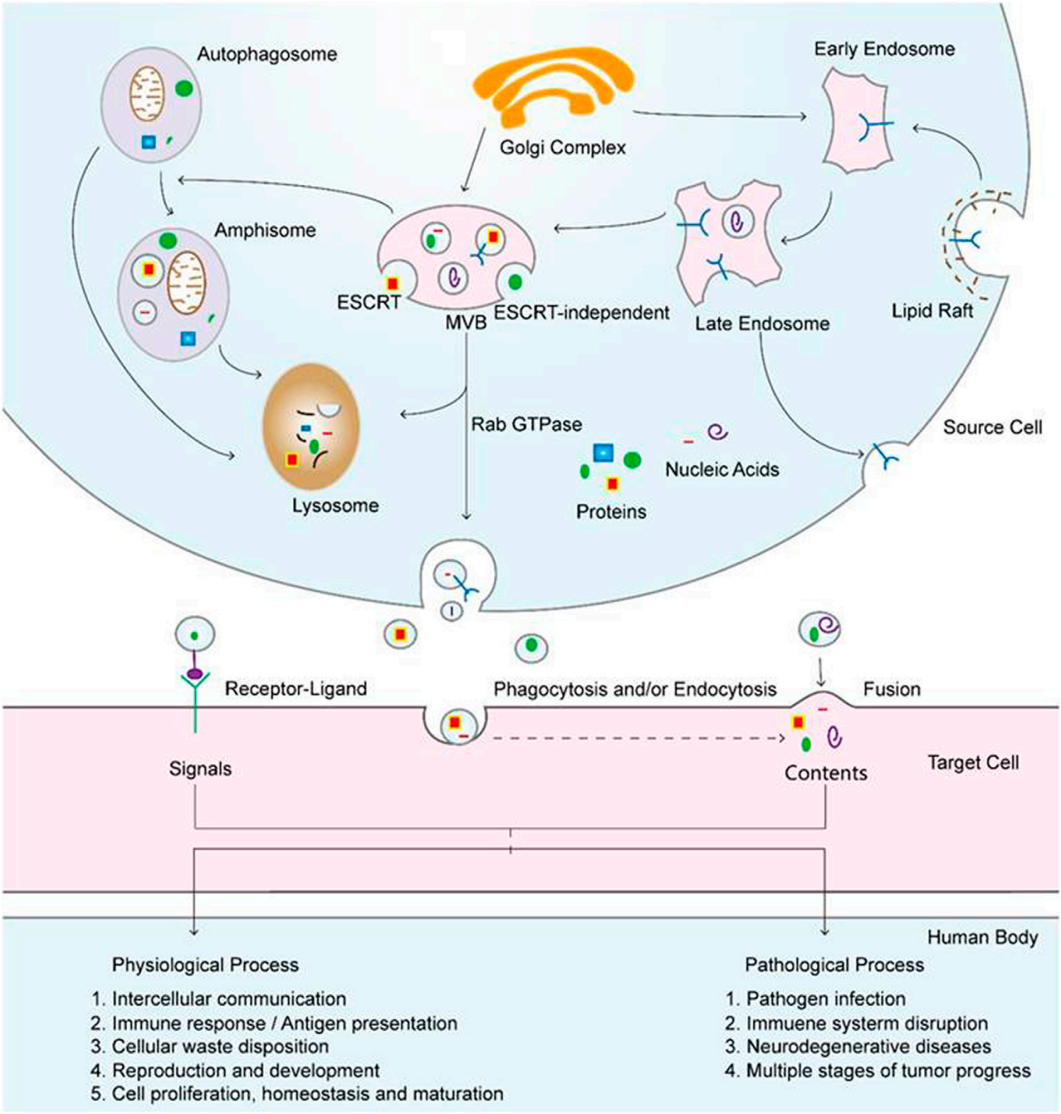
Exosomal biogenesis and internalization mechanisms and their roles in physiological and pathological processes. Exosomes are formed by inward budding from the endosomal membrane, which leads to the formation of multivesicular bodies (MVBs). MVBs can be fated for lysosomal degradation or fusion with the plasma membrane, which is associated with the release of exosomes. In addition, MVBs also participate in autophagosome maturation as endocytic fusion partners that meet with autophagosomes. Target cells internalize exosomes by three methods, which can facilitate the signaling and content delivery from source to target cells, thus mediating the progression of many physiological and pathological processes. Reproduced with permission of Ivyspring International Publisher (He et al., 2018).

Exosomes are a class of membrane-bound EVs that are 30–100 nm in diameter and are released by various cell types in the body. They can be found in a variety of body fluids, such as plasma, urine, and synovial fluid (Doyle and Wang, 2019). They contain diverse biomolecules, such as lipids, proteins, and nucleic acids (He et al., 2018). They act as vehicles to transfer biological signals encoded in proteins, lipids, mRNAs and miRNAs enclosed within a lipid bilayer between cells in autocrine and paracrine manners during both physiological and pathological processes (Connor et al., 2019). They are involved in many biological processes, such as cancer progression, immune response, cell proliferation, cell migration and angiogenesis (Than et al., 2017). They can be used as biomarkers, vaccines, drug delivery devices, and therapeutic tools (Raposo and Stoorvogel, 2013). As a new cell-free treatment, exosomes avoid the drawbacks of direct use of stem cells, such as phenotypic drift and tumor formation. In tendon tissue engineering, they can increase collagen synthesis and angiogenesis to remodel and prevent ECM degradation. Exosomes secreted from ADSCs can attenuate the early tendon inflammatory response after injury by inhibiting macrophage NF‐κB signaling coupled with reducing proinflammatory cytokine IIIb and the major collagenase Mmp-1 expression (Shen et al., 1722020). In addition to their function in inflammatory regulation, exosomes can also facilitate tendon healing by improving collagen production and organization and reducing scar formation (Chamberlain et al., 2021) (Figure 5).

Although exosomes have attracted considerable interest for tissue engineering, the functional mechanisms are still not clear. Despite their promising potential, it is difficult to isolate large quantities of pure and specific exosomes from mixtures of different vesicle types in a large volume of solution due to the technical challenges, costs, and lack of suitable biomarkers for particular exosomes (He et al., 2018). To further enrich exosomal content, multiple isolation methods have been used consecutively (Li et al., 2017). In addition, selecting the optimal administration route for effective and targeted biodistribution in specific contexts remains a challenge (Alqurashi et al., 2021). Moreover, the clinical use of exosomes is restricted due to the lack of standardization in exosome isolation and analysis methods (Doyle and Wang, 2019).

### 7.3 Platelet-rich plasma PRP

PRP is blood plasma with a high concentration of platelets obtained by removing red blood cells through the centrifugation of whole blood. PRP contains various growth factors, including PDGF, IGF-1, FGF and EGF, that can promote tendon repair by promoting cell recruitment, proliferation, and angiogenesis (Chen et al., 2018). In addition to various growth factors, activated PRP can also act as a 3-dimensional bioactive scaffold (fibrin gel) with a mesh-like microstructure for recruitment, proliferation, angiogenesis and incorporation (Yuan et al., 2013). PRP can be applied in the treatment of tendon injury because of its ability to regulate fibrosis and angiogenesis. PRP can promote the proliferation and differentiation of cells (fibroblasts, tendon stem/progenitor cells, and circulation-derived stem cells) and can enhance collagen (type I and type III) production (Zhang and Wang, 2010b). PRP therapy is considered safe because it has an autologous nature and exhibits long-term clinical effects without any reported major side effects (Middleton et al., 2012). In addition, the preparation method has been greatly simplified in recent years, which further facilitates clinical application (Yuan et al., 2012). However, injection is the main delivery method to treat tendon injury. A “one-size-fits-all” treatment regimen cannot satisfy the multiple injury models. Thus, exploring different delivery methods is a future study direction (Nakama et al., 2005; Jiang and Wang, 2013). Understanding the complex relationships among various molecules in PRP and selecting the ones with healing potential are vital measures to facilitate tendon repair.

### 7.4 Gene therapy

Gene therapy is a promising treatment method in which gene vectors are used to introduce a foreign nucleic acid (such as DNA or RNA) into a specific cell (Patil et al., 2019). The introduced gene sequences can persistently promote the healing response and restore tendon function before injury as completely as possible by producing signaling molecules and transcription factors and by further mediating the production of related proteins. The persistent effect of gene therapy avoids the drawbacks of the short half-lives of stem cells and cell-secreted products. Gene vectors are classified into viral and non-viral. Viral vectors are more efficient in the transfer of DNA to cells than non-viral vectors. However, viral vectors are more pathogenic because they contain viral proteins. There are two main vector transfer methods: directly transferring the vectors to the injured site and transferring genes to cells for cultivation *in vitro*, followed by transplanting to the target site. Compared with transferring genes to cells *in vitro*, direct gene transfer is less invasive and technically easier except for the disadvantage of inducing non-specific infection to adjacent cells (Huang et al., 2006). The indirect *in vitro* technique is safer because viral DNA is not administered directly to the host cells. The identification of optimal cellular targets, therapeutic genes and delivery vectors is an issue to resolve. Genomic profiling and screening were applied to detect tendon formation- and regeneration-related genes.

## 8 Other key elements in tendon tissue engineering

### 8.1 Mechanical loading and bioreactors

The responses of tendon cells to mechanical stimuli are critical to maintain tendon development, homeostasis, and regeneration after injury (Lavagnino et al., 2015). Understanding the relationship between mechanical stimuli and biochemical signals is crucial to identifying strategies and potential treatments for tendon repair (Nourissat et al., 2015). Appropriate mechanical stimuli can enhance the metabolism, structural and mechanical properties of tendons by increasing the collagen type I production of fibroblasts and the cross-sectional area and tensile strength of the tendons (Wang, 2006). Cyclic uniaxial mechanical stretch, similar to the mechanical environment in tendon tissues *in vivo*, can increase ECM production and organize collagen fibril alignment. Inappropriate mechanical stimuli can lead to tendon injury by increasing the production of inflammatory mediators, such as prostaglandin E2 (PGE2) and leukotriene B4 (LTB4) (Khan and Maffulli, 1998; Li et al., 2004). Cyclic mechanical longitudinal strain can stimulate proliferation and apoptosis, in contrast to extended stress duration. A constant stress duration can inhibit cell proliferation and apoptosis through increased HSP72 activity (Barkhausen et al., 2003).

Bioreactors provide a controlled environment for the systematic study of specific biological, biochemical, and biomechanical requirements for the design and manufacture of engineered tendon/ligament tissue. The strain environment constructed by bioreactors can mimic the *in vivo* physiological conditions of native tissue. Thorfinn et al. (2012) demonstrated that engineered tendons processed by bioreactors showed greater cellularity and strength than tendons cultured statically. However, the currently produced tissue constructs by bioreactors cannot yet mimic the intricate ECM remodeling process of native tendon tissue. To enhance the remodeling process, a future fabricated bioreactor with specific mechanical conditions is necessary to generate improved tissue grafts (Benhardt and Cosgriff-Hernandez, 2009).

### 8.2 Role of macrophage polarization in tendon repair

The immune system plays a central role in each tendinopathy stage and regulates the processes of tissue repair by immune cells and the secreted cytokines (Millar et al., 2017). Immune cells, including neutrophils, mast cells, monocytes/macrophages, B cells, CD4^+^ and CD8^+^ T cells, natural killer (NK) cells, and innate lymphoid cells, involved in the inflammation and regeneration processes of tendon injury. Different immune cells together with secreted immune modulators participate to control and promote tissue regeneration (Russo et al., 2022a). Thereinto, macrophages are the most studied immune cells with a crucial role in tendon injury and repair process.

As essential components of the innate immune response, macrophages play an important role in balancing inflammatory responses and coordinating the various processes of tissue repair and regeneration. Macrophages can remove necrotic debris by phagocytosis in the early stage of tendon healing and can secrete various growth factors, cytokines, and degrading enzymes to promote fibroblast/tenocyte proliferation and induce ECM synthesis and remodeling (Lin et al., 2018). Macrophages can be divided into two different cell phenotypes, M1 and M2 (Hou et al., 2021). M1 macrophages initiate the inflammatory response and perform antimicrobial activities with upregulated expression levels of proinflammatory factors, such as TNF-a and IL-1b (Lin et al., 2018). M2 macrophages initiate the repair process and the resolution of the inflammatory response by stabilizing angiogenesis, promoting fibroblast proliferation, and coordinating the ECM (Yue et al., 2015; de Torre-Minguela et al., 2016). M1 macrophages usually shift toward M2 macrophages within 2 weeks after the initiation of inflammation (Marsolais et al., 2001). The chronological appearance of M1 and M2 macrophages in the tissue repair process is the basis for regulating changes in the macrophage phenotype for tissue regeneration. In the early stage of tissue repair, M1 macrophages must be present at a reasonable level to promote tissue healing with a controlled inflammatory response. In the late stage, a reasonable level of M2 macrophages is beneficial for tissue stability and maturation. Thus, how to precisely control the macrophage switching pattern during tendon healing to promote repair outcomes is an important issue to resolve.

### 8.3 Inflammation

Maintaining the physiological homeostasis of tendon is the vital issue to guarantee to its function. Stromal, immune-sensing, and infiltrating compartments represent three different cell compartments that contribute each to a complex milieu of tendon homeostasis. The crosstalk between the host tissue, stem and immune cells might greatly modulate the immune reaction resolving hence the inflammatory response and avoiding the formation of fibrotic scar tissue (Russo et al., 2022b). Disruption of this balance results in tendon inflammation, tendinopathy or tendon injury (Chisari et al., 2019). Tendon injuries are accompanied by inflammation response. Inflammation plays an important role in the pathogenesis of tendon pathologies, in particular before clinical evidence of the condition (D’Addona et al., 2017), and is considered to be an essential process in the resolution of tendon healing.

As the initial stage of tendon healing after an acute injury, the inflammatory phase is characteristic of granulation formation with inflammatory cells infiltration and platelet activation. During the inflammation response process, various mediators include pro-inflammatory and anti-inflammatory cytokines and several growth factors including TNF-α, IL-1β, IL-6, IL-10, VEGF, TGF-b and 25 COX-2 and PGE2 are expressed by tenocytes and involved in the repair process (Chisari et al., 2019). As the inflammatory phase is the vital foundation of the next proliferative phase and remodelling phase, disruption of the inflammatory phase can result in tendon pathologies. However, the prolonged chronic inflammation process acts adverse effects on the tendon healing process (Chisari et al., 2021). And that, the excessive inflammatory responses and associated matrix metalloproteinases activities can also lead to scarlike tendon healing or pathology (Tarafder et al., 2017). Thus, we should keep the inflammatory response within a certain range of intensity and duration to promote tendon repair.

Till now, there have been some literatures published addressing regulating inflammation response to promote tendon injury repair. Some researchers studied the effects and mechanisms of medicines on tendon inflammation and have received positive results (Wang et al., 2019b; Wang et al., 2022). However, some other researchers provided different experiment results. For example, Heinemeier et al., found that 1-week treatment with ibuprofen elicited no change in adult human chronic tendinopathic tissues (Heinemeier et al., 2017), while Bittermann et al. (2018) showed that ibuprofen treatment during the inflammatory phase actually blunted healing in a model of murine Achilles tendinopathy. Except medicine, the interplay of MSCs with the tendon niche is also essential for the modulation of the inflammatory response following injury (Vinhas et al., 2018). As the resident cells of tendon, TDSCs participate in the regulation of inflammation during healing of acute tendon injuries. Connective tissue growth factor (CTGF) enriched CD146+ TDSCs were shown to reduce pro-inflammatory M1 cells in the early healing phase and express anti-inflammatory IL-10 and TIMP-3 *via* JNK/signal transducer and activator of transcription 3 (STAT3) signaling (Tarafder et al., 2017). It has been hypothesized before and demonstrated now that the effects of MSCs and soluble factors on inflammation may be dominated by paracrine action of MSCs. The study that conditioned medium derived from the culture of horse amniotic cells exerting both *in vitro* immunomodulatory potential and also facilitating the healing process in damaged tendons favored the perspective of paracrine action (Lange-Consiglio et al., 2013b). However, the underlying mechanism should be further studied. Furthermore, metabolic diseases, including obesity, diabetes, hyperlipidemia, *etc.*, can have negative influences on repair process by disrupting tendon homeostasis (Speed, 2016). Thus, dealing with the metabolic diseases is critical to promote the restoration of normal tendon structure and function.

In all, we may concluded that inflammatory plays an double-edged effects on tendon injury repair. Inflammation initiates the healing process and is also associated with the chronic tendinopathies. How to effectively control inflammatory response is vital to the tendon healing process. Further studies should be focused on tendon homeostasis, pathogenesis of tendinopathy, inflammation pathways, the interplay between different cell types, cellular and molecular targets to develop new innovative strategies to deal with inflammation.

## 9 Current challenges and future directions

Tissue engineering strategies aim to recapitulate the structure and function of the native tendon by biomimetic approaches. Although we have made some encouraging progress in the field of tendon tissue engineering, we still cannot completely regenerate tendon tissues with the complex composition and structure and the excellent mechanical functions of native tendons. The unsatisfactory results have prompted researchers to explore new biomaterials, fabrication technologies, cell types, biological adjuncts and other possible influencing factors to promote tendon regeneration. However, some key challenges remain to be overcome in reconstructing damaged tendon tissues.

The architecture of the scaffold is associated with the mechanical properties and biological response and can further determine the long-term clinical effects. Therefore, selecting an appropriate preparation method to fabricate suitable scaffolds with porous structures and excellent mechanical properties for tendon tissue engineering is an important issue. Fiber-based scaffolds fabricated by different methods have been the preferred option for tendon tissue engineering with the properties of mimicking the collagen fibers of the native tendon and promoting cell proliferation and collagen matrix deposition. Future scaffolds should satisfy the following requirements: simple and economical fabrication techniques, excellent structure mimicking the hierarchical structure of the native tendon, and good mechanical properties mimicking those of the native tendon, good biocompatibility, and a controllable degradation rate consistent with the tendon repair process.

Due to their easy availability, active metabolism and easy tendon lineage differentiation, MSCs are the most widely used cell type in tendon tissue engineering and have obtained favorable results. PSCs have also been applied in tendon tissue engineering; however, they are limited by the disadvantages of ethical concerns and teratoma or ectopic bone formation. AMSCs have presented the effects for tendon repair by the properties of modulating and suppressing inflammatory responses combined with the angiogenic, cytoprotective, anti-scarring, and antibacterial potential. The application of tenocytes and fibroblasts, the two main differentiated cell types, is frustrated by the disadvantages of being difficult to obtain and expand and their low activity rate. More attention should be devoted to obtaining knowledge about cell differentiation and signaling pathways and acquiring new culture techniques for cell purification and expansion. The combination of different cell types may be a desirable approach for tendon tissue engineering (Ruiz-Alonso et al., 2021). And that, cell-material interactions are also a key area of research. The fabricated scaffolds combined with bioactive motifs may provide new possibilities for material-regulated cell behavior to facilitate tendon repair or regeneration.

Cell products (growth factors, exosomes), PRP, gene therapy, mechanical stimuli, macrophage polarization and inflammation modulation can be combined with scaffolds and cells in different forms to promote repair. Cell products are characterized by diverse biological activities, including increasing cell proliferation, inducing stem cell differentiation to tenocytes and promoting ECM synthesis. Nevertheless, there are some limitations in the application of the cell products that need to be resolved, such as how to deliver the cell products into the body, how to ensure the continuous and controlled release of the cell products, and how to maintain the biological activity throughout the repair process. Gene therapy can avoid the disadvantages of direct use of cell products, making it a promising study direction. How to effectively make use of mechanical stimuli in the whole tendon repair process is also a vital issue to study. Furthermore, immune and inflammation modulation is another hot topic to research by the important role in tendon injury and repair process.

## 10 Conclusion

Considerable progress has occurred in all elements associated with tendon tissue engineering, and the field shows promising prospects for further research. Various biomaterials involving natural and synthetic polymers have been used in scaffolds and have shown excellent biocompatibility, biodegradability and biomechanics. Scaffolds with various compositions and structures mimicking the hierarchical architecture of the native tendon have been fabricated, and different cell types, cell products and other key factors have been incorporated to facilitate tendon repair and regeneration. Although some exciting progress has been made in tendon tissue engineering with regard the underlying mechanisms and experimental techniques *in vitro* or in animal models, much more progress is needed to enable further clinical application.

## Glossary

ECM: Extracellular matrix
TSCs: Tendon stem cells
MSCs: Mesenchymal stem cells
LLLT: Low-level laser therapy
ALG: Alginate
HA: Hyaluronic acid
CS: Chitosan
CS/GE-TA: Chitosan/gelatin-tannic acid
ZnO: Zinc oxide
SF: Silk fibroin
PPDO: Poly(p-dioxanone)
PGA: Polyglycolic acid
PLA: Polylactic acid
PLGA: Polylactic-co-glycolic acid
PCL: Polycaprolactone
PDGF-BB: Platelet-derived growth factor BB
E-jetting: Electrohydrodynamic jet printing
PLACLAC: Poly (L-lactide-co-Ɛ-caprolactone-co-acryloyl carbonate)
SEM: Scanning electron microscopy
PSCs: Pluripotent stem cells
ESCs: Embryonic stem cells
PRP: Platelet-rich plasma
TSPCs: Tendon stem/progenitor cells
Scx: Scleraxis
Tnmd: Tenomodulin
3-D: Three-dimensional
Thbs4: Thrombospondin 4
CTGF: Connective tissue growth factor
IGF-I: Insulin-like growth factor 1
TGF-β: Transforming growth factor-β
VEGF: Vascular endothelial growth factor
bFGF: Basic fibroblast growth factor
PGE2: Prostaglandin E2
LTB4: Leukotriene B4
